# Supplementary material for: Utilisation of tools to facilitate cross-border communication during international food safety events, 1995–2019: a realist synthesis protocol
Source: BMJ Open. 2019 Oct 28;9(10):e030593. doi: 10.1136/bmjopen-2019-030593 (PMC6830981; doi:10.1136/bmjopen-2019-030593)
Supplement: Supplementary data [file bmjopen-2019-030593supp001.pdf]

Supplementary File 1 – Preliminary inventory of communication tools currently used to exchange information during international food safety events*

Name of tool	Description	Type of Users	Primary Mode of Communication	Primary type of information exchanged	Coordinating Responsibly
International Food Safety Authorities Network<br><br>(INFOSAN)	Rapid sharing of information during food safety emergencies to stop the spread of contaminated food from one country to another; facilitates the sharing of experiences and tested solutions in and between countries in order to optimise future interventions to protect the health of consumers; launch in 2004	Officially designated government officials from multiple sectors involved in food safety (e.g. health, agriculture, veterinary services, trade, etc.); Members from 186 countries + European Commission (European Centre for Disease Control (ECDC), European Food Safety Authority (EFSA), Rapid Alert System for Food and Feed (RASFF) Commission Contact Point)	Web-based communication platform<br><br>(INFOSAN Community Website, members only)	Food contamination information along with export details; foodborne outbreak information including related epidemiologic, laboratory and traceability information	Secretariat at WHO; (Jointly managed by FAO and WHO)
For more information, see: http://www.who.int/foodsafety/areas_work/infosan/en/					
Rapid Alert System for Food and Feed<br><br>(RASFF)	Provides food and feed control authorities with an effective tool to exchange information about measures taken responding to serious risks detected in relation to food or feed; launched in 1979	Members from 28 national food safety authorities in the EU + EFSA, ESA, Norway, Liechtenstein, Iceland and Switzerland & WHO	Web-based communication platforms (RASFF Window, members only)	Food contamination information along with export details; foodborne outbreak information including related epidemiologic, laboratory and traceability information	Directorate General for Health and Food Safety<br><br>(DG SANTE, European Commission)
For more information, see: https://ec.europa.eu/food/safety/rasff_en					
Early Warning and Response system of the European Union<br><br>(EWRS)	Confidential computer system allowing Member States to send alerts about events with a potential impact on the EU, share information, and coordinate their response	Members from 28 Public Health Authorities of the EU Member States, which have been designated officially by the Government of their Country as members of the EWRS, and the competent service of the European Commission & WHO	Web-based communication platform (members only)	Foodborne disease surveillance information	ECDC<br><br>(European Commission)
For more information, see: http://www.eurosurveillance.org/ViewArticle.aspx?ArticleId=666					
Epidemic Intelligence Information System for Foodborne and Waterborne diseases and Zoonoses<br><br>(EPIS-FWD)	Facilitates the early detection and assessment of multi-country/multinational molecular typing clusters and outbreaks of Foodborne and Waterborne diseases and Zoonoses	The platform connects epidemiologists and microbiologists from 52 countries: 28 EU Member States, three countries of the European Economic Area (EEA) - Iceland, Norway and Liechtenstein - and 21 other non-EU countries & WHO	Web-based communication platform (members only)	Molecular typing clusters and foodborne, waterborne and Zoonotic outbreak details;	ECDC (European Commission)
For more information, see: http://ecdc.europa.eu/en/aboutus/what-we-do/epidemic-intelligence/Pages/EpidemicIntelligence_Tools.aspx					
Hepatitis A Lab-Network	Global network of hepatitis A reference laboratories	Scientists working in hepatitis A reference laboratories	Email; Web-based electronic database (members only)	Molecular and epidemiological data on hepatitis A virus	National Institute for Public Health and the Environment

(HAVNet)					(RIVM, The Netherlands)
For more information, see: http://www.eurosurveillance.org/ViewArticle.aspx?ArticleId=21356					
PulseNet International	Network of national and regional laboratory networks dedicated to tracking foodborne infections world-wide; Each laboratory utilises standardised genotyping methods, sharing information in real-time	Scientists working in public health laboratories and academic and medical institutions, including 10 countries in the Eastern Mediterranean, 16 countries in the Americas, 11 countries in the Western Pacific, 11 countries in Africa, 31 countries in Europe	PulseNet International forum hosted by PulseNet Canada (Web-based, members only, communication platform)	Genotyping information from foodborne pathogens (some genomic data)	US Centers for Disease Control (CDC)
For more information, see: http://www.pulsenetinternational.org/					
ASEAN Rapid Alert System for Food and Feed	Regional network modelled after the European RASFF system involving 10 ASEAN Member States.	Competent Authorities in Food Safety and Public Health of all ASEAN Member States (10 countries)	web-based application	Information on direct or indirect risks to human deriving from food or feed being traded in ASEAN and measures taken to prevent them entering the food chain	National Bureau of Agricultural Commodity and Food Standards (ACFS),<br><br>Ministry of Agriculture and Cooperatives, Thailand.
For more information, see: http://arasff.net/index.php					
Bi-lateral agreements	Many national authorities have established bi-lateral agreements with foreign agencies for the purpose of exchanging information during international food safety events.	National authorities.	Email or telephone.	Could be any type of information required to respond to an international food safety event.	National authorities.

*This list has been informed by the author's experience as the WHO secretariat of INFOSAN, the results from a scoping search, and input from the Expert Reference Committee
