# Supplementary material for: Utilisation of tools to facilitate cross-border communication during international food safety events, 1995–2019: a realist synthesis protocol
Source: BMJ Open. 2019 Oct 28;9(10):e030593. doi: 10.1136/bmjopen-2019-030593 (PMC6830981; doi:10.1136/bmjopen-2019-030593)
Supplement: Supplementary data [file bmjopen-2019-030593supp002.pdf]

Supplementary file 2 - Definitions of key terms

Communication tools: Networks, knowledge sharing platforms, technical programmes, or systems that facilitate communication related to food safety across national borders.

Context: The context includes the social, cultural, institutional, historical and environmental factors that form the setting in which actions are taken to trigger mechanisms.

Context–mechanism–outcome configuration: A relationship between the elements of realist synthesis; how mechanisms are triggered under specific contexts to result in outcomes.

International Food Safety Events: An international food safety event results when unsafe food produced in one country, is exported to at least one other country (and may or may not result in an outbreak of foodborne illness).

Mechanism: Mechanisms can be understood as the underlying context-dependent processes, behaviours, structures, values or levers that are able to generate outcomes.

Outcome: The impact or behaviours resulting from the interaction between mechanisms and contexts.

Programme theory: A set of theoretical explanations or assumptions about how a particular programme, process or intervention is expected to work or cause the intended or observed outcomes.
