# Supplementary material for: Utilisation of tools to facilitate cross-border communication during international food safety events, 1995–2019: a realist synthesis protocol
Source: BMJ Open. 2019 Oct 28;9(10):e030593. doi: 10.1136/bmjopen-2019-030593 (PMC6830981; doi:10.1136/bmjopen-2019-030593)
Supplement: Supplementary data [file bmjopen-2019-030593supp003.pdf]

Supplemental File 3 – Detailed Search Strategies for Web of Science, Embase, MEDLINE, PubMed and CINAHL

Web of Science

Search History:

Set	Results	Save History / Create Alert Open Saved History
# 7	1,027	#6 AND #5 AND #4 *Indexes=SCI-EXPANDED, SSCI, A&HCI, CPCI-S, CPCI-SSH, ESCI Timespan=1995-2019*
# 6	9,703,534	TS=(systems OR network OR tool OR communication OR notification OR “information exchange”) *Indexes=SCI-EXPANDED, SSCI, A&HCI, CPCI-S, CPCI-SSH, ESCI Timespan=1995-2019*
# 5	806,837	TS=(international OR multi-state OR multi-country OR imported OR exported) *Indexes=SCI-EXPANDED, SSCI, A&HCI, CPCI-S, CPCI-SSH, ESCI Timespan=1995-2019*
# 4	32,199	#3 OR #2 OR #1 *Indexes=SCI-EXPANDED, SSCI, A&HCI, CPCI-S, CPCI-SSH, ESCI Timespan=1995-2019*
# 3	26,514	TS=("food safety" OR "food contamination" OR "foodborne diseases") *Indexes=SCI-EXPANDED, SSCI, A&HCI, CPCI-S, CPCI-SSH, ESCI Timespan=1995-2019*
# 2	1,793	TS=(gastroenteritis NEAR/5 (incident* OR emergenc* OR outbreak*)) *Indexes=SCI-EXPANDED, SSCI, A&HCI, CPCI-S, CPCI-SSH, ESCI Timespan=1995-2019*
# 1	5,547	TS=(food* NEAR/5 (incident* Or emergenc* OR outbreak*)) *Indexes=SCI-EXPANDED, SSCI, A&HCI, CPCI-S, CPCI-SSH, ESCI Timespan=1995-2019*

Embase and MEDLINE

☐	# ▲	Searches	Results
☐	1	▶ (food* adj5 incident*).af.	983
☐	2	▶ (food* adj5 emergenc*).af.	5617
☐	3	▶ (food* adj5 outbreak*).af.	8825
☐	4	▶ (gastroenteritis adj5 incident*).af.	54
☐	5	▶ (gastroenteritis adj5 emergenc*).af.	1206
☐	6	▶ (gastroenteritis adj5 outbreak*).af.	4424
☐	7	▶ ("food safety" or "food contamination" or "foodborne diseases").af.	156116
☐	8	▶ (international or multi-state or multi-country or imported or exported).af.	3541525
☐	9	▶ (systems or network or tool or communication or notification or information-exchange).af.	4810679
☐	10	▶ 1 or 2 or 3	15272
☐	11	▶ 4 or 5 or 6	5640
☐	12	▶ 7 or 10 or 11	170450
☐	13	▶ 8 and 9 and 12	2835
☐	14	▶ limit 13 to yr="1995 -Current"	2779

PubMed

Search	Add to builder	Query	Items found	Time
#7	Add	Search **((((systems OR network OR tool OR communication OR notification OR “information exchange”))) AND ((international OR multi-state OR multi-country OR imported OR exported))) AND (((((("food safety" OR "food contamination" OR "foodborne diseases"))) OR ((gastroenteritis AND (incident* OR emergenc* OR outbreak*)))) OR ((food* AND (incident* Or emergenc* OR outbreak*))))**	1062	10:27:02
#6	Add	Search **(systems OR network OR tool OR communication OR notification OR “information exchange”)**	2269385	10:26:36
#5	Add	Search **(international OR multi-state OR multi-country OR imported OR exported)**	528235	10:26:03
#4	Add	Search **(((("food safety" OR "food contamination" OR "foodborne diseases"))) OR ((gastroenteritis AND (incident* OR emergenc* OR outbreak*)))) OR ((food* AND (incident* Or emergenc* OR outbreak*)))**	105011	10:25:24
#3	Add	Search **("food safety" OR "food contamination" OR "foodborne diseases")**	74378	10:24:58
#2	Add	Search **(gastroenteritis AND (incident* OR emergenc* OR outbreak*))**	13879	10:24:38
#1	Add	Search **(food* AND (incident* Or emergenc* OR outbreak*))**	26356	10:24:21

CINAHL (with proximity operators; syntax: n5)

	Search ID#	Search Terms	Search Options	Actions
☐	S7	S4 AND S5 AND S6	**Search modes** - Find all my search terms	**View Results** (79) **View Details** **Edit**
☐	S6	systems OR network OR tool OR communication OR notification OR "information exchange"	**Search modes** - Find all my search terms	**View Results** (836,810) **View Details** **Edit**
☐	S5	international OR multi-state OR multi-country OR imported OR exported	**Search modes** - Find all my search terms	**View Results** (125,224) **View Details** **Edit**
☐	S4	S1 OR S2 OR S3	**Search modes** - Find all my search terms	**View Results** (6,148) **View Details** **Edit**
☐	S3	"food safety" OR "food contamination" OR "foodborne diseases"	**Search modes** - Find all my search terms	**View Results** (5,155) **View Details** **Edit**
☐	S2	gastroenteritis N5 (incident* OR emergenc* OR outbreak*)	**Search modes** - Find all my search terms	**View Results** (417) **View Details** **Edit**
☐	S1	food* N5 (incident* Or emergenc* OR outbreak*)	**Search modes** - Find all my search terms	**View Results** (889) **View Details** **Edit**
