# Supplementary material for: Utilisation of tools to facilitate cross-border communication during international food safety events, 1995–2019: a realist synthesis protocol
Source: BMJ Open. 2019 Oct 28;9(10):e030593. doi: 10.1136/bmjopen-2019-030593 (PMC6830981; doi:10.1136/bmjopen-2019-030593)
Supplement: Supplementary data [file bmjopen-2019-030593supp004.pdf]

Supplemental File 4 - Data Extraction Form

**EndNote Reference Number**
**Title**
**Authors**
**Year of publication**
**Type of document/study**
**Countries involved (specify where food was produced, where food was distributed, and where illnesses occurred)**
**International/regional organizations involved (specify who took a coordinating role)**
**Specific foodborne hazard**
**Implicated food item (specify how food was implicated, e.g. epidemiologic evidence, laboratory evidence, traceability evidence)**
**Name and details of communication tool(s) used (including who used the tool and what kind of information was exchanged)**
**Factors that facilitated the use of the tools (context or mechanisms)**
**Factors that limited the use of the tool (context or mechanisms)**
**Conclusions made by the authors with respect to the use of the tools (related to context, mechanisms or outcomes)**
**Recommendations made by the authors with respect to improving international communication during international food safety events**
**Any other contextual factors**

**Any other underlying mechanisms**
**Points of discussion to raise with Expert Reference Committee**
