# Supplementary material for: Utilisation of tools to facilitate cross-border communication during international food safety events, 1995–2019: a realist synthesis protocol
Source: BMJ Open. 2019 Oct 28;9(10):e030593. doi: 10.1136/bmjopen-2019-030593 (PMC6830981; doi:10.1136/bmjopen-2019-030593)
Supplement: Author's manuscript [file bmjopen-2019-030593.draft_revisions.pdf]

BMJ Open is committed to open peer review. As part of this commitment we make the peer review history of every article we publish publicly available.

When an article is published we post the peer reviewers' comments and the authors' responses online. We also post the versions of the paper that were used during peer review. These are the versions that the peer review comments apply to.

The versions of the paper that follow are the versions that were submitted during the peer review process. They are not the versions of record or the final published versions. They should not be cited or distributed as the published version of this manuscript.

BMJ Open is an open access journal and the full, final, typeset and author-corrected version of record of the manuscript is available on our site with no access controls, subscription charges or pay-per-view fees (http://bmjopen.bmj.com).

If you have any questions on BMJ Open's open peer review process please email info.bmjopen@bmj.com

BMJ Open

Utilisation of tools to facilitate cross-border communication during international food safety events, 1995-2019: A realist synthesis protocol

Journal:	*BMJ Open*
Manuscript ID	bmjopen-2019-030593
Article Type:	Protocol
Date Submitted by the Author:	21-Mar-2019
Complete List of Authors:	Savelli, Carmen; Organisation mondiale de la Sante, Food Safety and Zoonoses; Lancaster University Faculty of Health and Medicine, Health Research<br>Mateus, Ceu; University of Lancaster, Division of Health Research
Keywords:	Realist review, contextual factors, food safety, foodborne disease outbreaks, international networks, communication tools

SCHOLARONE™
Manuscripts

Title: Utilisation of tools to facilitate cross-border communication during international food safety events, 1995-2019: A realist synthesis protocol

Authors: Carmen Joseph Savelli[1,2], Céu Mateus[2]

1) World Health Organization, Geneva, Switzerland
2) Lancaster University, Lancaster, United Kingdom

Corresponding Author: Carmen Joseph Savelli
World Health Organization
Food Safety and Zoonoses
Avenue Appia 20
1211, Geneva, Switzerland
savellic@who.int
+41 22 79 13234

Key words: realist review – contextual factors – food safety – foodborne disease outbreaks – international networks – communication tools

Word Count: 4604

For peer review only

For peer review only - http://bmjopen.bmj.com/site/about/guidelines.xhtml

Abstract

Introduction: Efficient communication and coordination between countries is needed for prevention, detection and response to international food safety events. While communication tools exist, current evidence suggests that they are only effective within certain contexts and only cover certain geographic areas. There is a need to unpack and explore the mechanisms of how and in what context such communication tools and their components are effective at facilitating international communication and coordination to keep food safe and mitigate the burden of foodborne disease around the globe.

Methods and analysis: A realist synthesis will be undertaken to understand how and why certain processes and structures of communication tools, utilised during international food safety events, influence their utility and effectiveness according to different contextual factors. The focus of this review is explanatory and aims to develop and test theory regarding how contextual factors trigger specific processes and mechanisms to produce outcomes. Using the realist context-mechanism-outcome configuration of theory development, a range of sources have been utilised to develop the initial programme theory, including the author's experience, a scoping review of published papers and grey literature, and input from an expert reference committee. To support, expand or refute the initial theory, data will be synthesised from published literature and input from the expert reference committee.

Ethics and dissemination: Ethical approval is not required for this review as it does not involve primary research. However, it will be conducted according to the appropriate ethical standards of accuracy, utility, usefulness, accountability, feasibility and propriety. The RAMESES publication standards will be followed to report the findings of this review. Upon completion, the final manuscript will be shared with members of the FAO/WHO International Food Safety Authorities Network (INFOSAN) and published in a peer reviewed journal.

Strengths and limitations of the study:

For peer review only - http://bmjopen.bmj.com/site/about/guidelines.xhtml

- Uses realist methods to explore contextual factors and underpinning causal mechanisms of complex international communication during food safety events.
- Utilization of an international expert review committee consisting of national government officials, international civil servants and academics to ensure complete coverage of the literature.
- Formulation of the context-mechanism-outcome programme theory relies heavily on published literature and therefore may be subject to publication bias.
- Review findings will be context -specific and therefore must be considered within the context of this research.

Systematic review registration: This protocol has been registered with PROSPERO, registration number: <<TBD after peer review>>

Background

Access to sufficient amounts of safe and nutritious food is a basic requirement for human health. However, around the world unsafe food is known to cause more than 200 acute and chronic diseases, ranging from diarrhoea to cancer.[1] In 2015, the first estimates of the global burden of foodborne diseases were reported by the WHO, indicating that 31 hazards (including bacteria, viruses, parasites, toxins and chemicals) were responsible for 600-million cases of foodborne diseases and 420,000 deaths worldwide in 2010.[2] This burden was disproportionately felt by children under five years of age who accounted for 40% of foodborne disease cases and 125,000 deaths.[2] While foodborne diseases are observed worldwide, Africa, South-East Asia and the Eastern Mediterranean regions report the highest burden.[2] In such high-burden areas, unsafe food presents additional consequences beyond disease burden, impeding socioeconomic development, overloading strained healthcare systems and damaging national economies, trade and tourism.[3] Furthermore, a 2018 study by the World Bank[4] indicates that unsafe food costs low- and middle-income economies approximately $100 billion USD in lost productivity and medical expenses each year.

For peer review only

For peer review only - http://bmjopen.bmj.com/site/about/guidelines.xhtml

Foodborne diseases are preventable, however prevention requires investment and coordinated action across multiple sectors to strengthen national food safety systems. Multiple agencies responsible for health, agriculture, veterinary services, trade, and several others must work together in order to build a strong and resilient national food safety system. The WHO has identified a number of core capacities that national governments should develop in order to safeguard national food supplies. The development of such core capacities are evaluated yearly by the WHO to determine whether or not countries have established functional mechanisms for the detection, prevention and response to foodborne disease and food contamination events. Data from 2017 indicate that 78% of the attributes of core capacities required for food safety have been developed globally, although disparities exist between regions. For example, 90% of the required core capacities have been achieved in Europe, while in Africa, only 54% of the core capacities have been achieved.[5]

An international food safety event results when unsafe food produced in one country, is exported to at least one other country. Recent international food safety events have demonstrated that even in countries with well-developed capacities related to food safety, unsafe foods that are produced abroad and imported for domestic consumption have the potential to result in large-scale outbreaks of foodborne disease. For example, nearly 4000 people became infected with *E.coli* (and nearly 800 developed haemolytic uremic syndrome) in Germany following the consumption of contaminated fenugreek sprouts, imported from Egypt in 2011. Illnesses related to the same imported product were concurrently reported in France.[6] In 2012, at least 11,000 cases of norovirus infection were reported in Germany following the consumption of frozen strawberries imported from China.[7] In 2008, 300,000 infants and children became ill in China, six of whom died, as a result of consuming milk products contaminated with melamine. The contaminated products were directly exported or secondarily distributed to 47 countries around the world.[8] In 2013 and 2014, nearly 1,500 cases of hepatitis A infection were identified in 13 European countries and linked to the consumption of internationally distributed frozen berries. [9] More recently, in 2017 and 2018 the world bore witness to the largest outbreak of listeriosis on record which occurred in South Africa and resulted in more than 1000 cases

For peer review only - http://bmjopen.bmj.com/site/about/guidelines.xhtml

and 200 deaths. This protracted outbreak was eventually linked to domestically produced ready-to-eat meat products which were exported to 15 other countries in Africa.[10] Also in 2017 and 2018, an outbreak of salmonellosis in France affecting 37 infants was linked to contaminated infant formula that was exported worldwide to more than 80 countries.[11] These examples represent some of the largest international food safety events that have occurred in the recent past, either in terms of case-counts or number of countries affected, but smaller-scale events occur on a regular basis. Furthermore, these events illustrate that even the most advanced food safety systems do not eliminate all foodborne risks from reaching the public. The globalisation of our food supply means that unsafe food produced in one country can certainly result in cases of foodborne disease abroad.

Global food trade grew almost threefold from 2005 to 2015[12] and is projected to continue to rise.[13] Thus, there is a need for international coordination to facilitate rapid and efficient communication and collaboration between public health and food safety authorities (i.e. competent authorities) worldwide to prevent, detect and respond to international food safety events when internationally traded food is deemed unsafe. Until relatively recently, timely mechanisms to facilitate such global communication did not exist. In the early 2000s, WHO Member States recognised this gap and adopted resolutions at the World Health Assemblies in 2000[14] and 2002[15] calling for improved communication and coordination during international food safety events, including better tools to facilitate this. Since then, advancements in communication technology have facilitated the development or expansion of international networks and knowledge sharing platforms to exchange molecular subtyping information of foodborne pathogens, epidemiologic information about foodborne diseases, as well as information on food contamination and related traceability details. Throughout this protocol, the term 'communication tool' will be used to encompass networks, knowledge sharing platforms, technical programmes, or systems that facilitate communication related to food safety across national borders. While various communication tools now exist (see supplementary file 1), evidence from practice suggests that such tools are only effective within certain contexts and only cover certain geographic areas. [16, 17, 18, 19]. It is therefore necessary to unpack and explore the

For peer review only - http://bmjopen.bmj.com/site/about/guidelines.xhtml

mechanisms of how and in what context such communication tools and their components are effective to facilitate international communication and coordination.

Unfortunately, a paucity of research has been conducted to investigate the attributes and effectiveness of the tools to facilitate cross-border communication during international food safety events. To date, most of the publications mentioning such tools focus on summarising a particular incident response, rather than explicitly examining the tools that were utilised. However, it is rather common for such reports of international food safety events to conclude by recommending that international efforts to strengthen rapid and efficient information exchange be improved through the further enhancement or utilisation of existing international networks and communication tools. Such reports also commonly refer to context specific factors that facilitate or prevent rapid communication on various aspects of food safety investigations, such as poorly developed food safety systems, lack of national coordination, or limited technical capacity. Available research provides limited guidance for decision-makers coordinating international programmes that facilitate information exchange on food safety, on how to adopt best practices to achieve their objectives. In addition, as explained by Savelli, Bradshaw, Ben Embarek and Mateus[20], the global food safety community would benefit from a thorough mapping of the interlinkages between such programmes and networks to better understand how they are being used, by whom and in what contexts. A realist synthesis is therefore proposed to begin to address this gap. The main question to guide this research is: how do different tools facilitate cross-border communication during international food safety events, why are they used, by whom, and for what purpose?

In this review, the proximal outcome of interest is the use of different tools to communicate internationally about issues related to food safety in an efficient manner. The distal outcomes of interest can be understood as the outcomes or consequences of using the tools. Some examples may include the identification of the source of an outbreak, facilitation of risk management actions in different countries, and prevention of foodborne disease. Although important, it is beyond the scope of this review to examine and measure the impact that using different tools has on the overall safety of

For peer review only - http://bmjopen.bmj.com/site/about/guidelines.xhtml

the global food supply. However, a number of insights relating to the utility of different tools to prevent or mitigate the burden of foodborne disease will be garnered from this review to be further explored in future research.

Methods/design

This synthesis will adhere to the 2005 protocol provided by Pawson, Greenhalgh, Harvey, and Walshe for conducting realist reviews and reporting will be guided by the Realist and Meta-narrative Evidence Synthesis: Evolving Standards (RAMESES) from Wong, Greenhalgh, Westhorp, Buckingham, and Pawson.[21] The five steps for conducting a realist review according to Pawson et al.[22] will be followed: 1) clarify scope; 2) search for evidence; 3) appraise primary studies and extract data; 4) analyse and synthesise evidence; and 5) disseminate. While presented sequentially, these steps are iterative and will be revisited throughout the review process when new evidence emerges that can contribute to theory refinement. The macro-level development theories that provide an overarching framework for this review include the third wave of modernisation theory developed in the 1990s [23, 24] and globalisation theory as articulated by Robinson.[25] Both theories provide a lens through which to understand that while the world is becoming ever more interconnected and interdependent, certain structures built to support development cannot be imposed in exactly the same way at the same time in different countries because the country-specific context will influence the outcomes. With this understanding and using the realist approach, middle-range programme theory will be developed to explain context-mechanism-outcome (C-M-O) configurations related to the use of communication tools to facilitate information exchange during international food safety events. Two reviewers will undertake this work and an expert reference committee will be established to provide feedback during the review, consisting of several coordinators of international communication tools currently in use. The review will be conducted over a six-month period from January 2019 to June 2019 (see Figure 1 for an overview of the stages of this review).

The realist approach

For peer review only - http://bmjopen.bmj.com/site/about/guidelines.xhtml

A realist approach has been chosen to conduct this review as it is well suited for the examination of complex programmes through its focus on outcomes in real-world settings and the contextual factors that influence them.[22] A realist perspective of social change underpins this approach whereby the actions of individuals and their understanding of the world serve to construct social phenomena and are influenced by cultural, institutional and social structures.[26, 27] This interpretative method is theoretically driven and allows evidence from a range of study designs to be synthesised. The use of theory facilitates a deeper understanding with respect to policy intentions and appreciates the complexity of programmes by including context in the analysis.[21] The overall intent of a realist review is the development of programme theories to understand how context influences mechanisms to generate outcomes. Mechanisms can be understood as the underlying context-dependent processes, behaviours, structures, values or levers that are able to generate outcomes. The context includes the social, cultural, institutional, historical and environmental factors that form the setting in which actions are taken to trigger mechanisms. The resulting outcomes of the programme, system or intervention under examination are the products of certain mechanisms being triggered in certain contexts and may be intended or unintended.[22, 28, 29]

In this review, identifying mechanisms will help to explain how competent authorities utilise existing communication tools during international food safety events in order to exchange information across national borders. Taking the realist perspective, several C-M-O configurations may be articulated within the programme theory to explain this phenomenon. The C-M-O configurations will allow the research to be abstracted and applied to multiple contexts, bolstering external validity. The process of theory building and configuring the C-M-O will be iterative, enabling the confirmation, refutation or modification of the initial programme theory.[21, 22, 30]

Research aim and objectives

The primary aim of this synthesis is to address the question: how do different tools facilitate cross-border communication during international food safety incidents, why are they used, by whom, and for what purpose? The overall objective is to refine a programme theory that explains the contexts

For peer review only - http://bmjopen.bmj.com/site/about/guidelines.xhtml

in which certain mechanisms generate certain outcomes by developing a series of C-M-O statements. This programme theory should prove useful to programme coordinators to promote and support the use of communication tools and improve their effectiveness. The specific objectives are as follows:

1) Document the different tools used to facilitate cross-border communication during international food safety incidents;
2) Examine the outcomes observed in relation to the use of different communication tools;
3) Identify and explain the mechanisms that influence the outcomes observed in relation to the use of different communication tools;
4) Identify the contextual factors that trigger mechanisms to influence the outcomes observed in relation to the use of different communication tools; and
5) Develop a realist programme theory that synthesises review findings and input from an expert reference committee to explain how different tools facilitate cross-border communication during international food safety events, why they are used, by whom, and for what purpose.

Identifying initial programme theory

To identify an initial programme theory, a range of sources have been utilised including the author's experiences as the current secretariat of the FAO/WHO International Food Safety Authorities Network (INFOSAN), a scoping review of published papers describing international food safety events and grey literature pertaining to various food safety communication tools currently in use and elicitation of input from an international expert reference committee. This preliminary work has proposed a C-M-O configuration to suggest that when the context is such that a country: 1) is an importer or exported of food commodities; 2) has the technical infrastructure to detect food safety events (including foodborne disease outbreaks or food contamination); and 3) is governed in accordance with regional and/or global laws and regulations relating to food control and global health security, then certain mechanisms including trust, experience, support, awareness, understanding and

For peer review only - http://bmjopen.bmj.com/site/about/guidelines.xhtml

a sense of community will facilitate the proximal outcome of using communication tools to relay information abroad and a potential range of distal outcomes, including: 1) intersectoral collaboration among different national stakeholders from agriculture, food and health authorities; 2) efficient exchange of information between international stakeholders; 3) timely detection, notification, and response to food safety events (including the implementation of risk management measures); 4) reduction of food safety risks; 5) robust understanding of the international dimensions of a given food safety event; and 6) prevention of foodborne disease. It is proposed that variations in the context will influence whether or not the proposed mechanisms will trigger the outcomes. A schematic overview of this initial programme theory is provided in Figure 2.

Search strategy

To test the initial programme theory, a systematic search of the literature will aim to identify documents written in English, dating back to 1995 that illuminate how different tools facilitate cross-border communication during international food safety events, why they are used, by whom, and for what purpose. This search will be undertaken using the databases Web of Science, Embase, MEDLINE, PubMed and CINAHL. A comprehensive search algorithm has been developed with assistance from a librarian at Lancaster University, United Kingdom, by first selecting key search terms following the review of titles and abstracts from 10 known publications describing international food safety events or an international food safety communication tool, system or network. Combinations of the following key words in English (and their truncations where required) using Boolean operators and proximity operators (where possible) will be entered into the selected databases: (systems OR network OR tool OR communication OR notification OR “information exchange”) AND (international OR multi-state OR multi-country OR imported OR exported) AND ((("food safety" OR "food contamination" OR "foodborne diseases") OR (gastroenteritis AND (incident OR emergency OR outbreak)) OR (food AND (incident Or emergency OR outbreak)))

For peer review only - http://bmjopen.bmj.com/site/about/guidelines.xhtml

Bibliographic references from documents selected for inclusion will be reviewed using the snowballing method to identify other potentially relevant documents. Since grey literature can be a pertinent source of information for realist reviews, annual reports, evaluation summaries, or policy documents published by international organisations or government agencies will also be searched for on respective websites.[22] The search for evidence will be driven by the research objectives and will be iterative in practice in order to identify all relevant information sources to develop the programme theory. Searching will conclude when theoretical saturation is reached and sufficient evidence has been collected to confidently assert that the proposed theory is plausible.[21] The expert reference committee will contribute to this research by identifying additional articles and documents for consideration in the review and will provide feedback on the emerging programme theories as they are developed. The search strategy will also be reviewed iteratively by this committee to ensure the scope of the search is appropriately designed to achieve the overall research aim and objectives. Throughout this process, references will be managed using Endnote X7 software.

Study selection criteria and procedures

To ensure that the development of programme theory considers a wide range of evidence, it is customary to utilise broad inclusion/exclusion criteria in a realist synthesis.[22] The inclusion criteria are studies of any design from peer-reviewed literature and other documents from grey literature that are written in English, published in 1995 or later, describe an international food safety event or a communication tool, and provide evidence that contributes to the synthesis and the emerging programme theory. The exclusion criteria are if a study does not describe an international food safety event or a communication tool with sufficient details to inform the programme theory, focuses on an outdated communication technology (e.g. facsimile), or is purely anecdotal. Two reviewers will independently screen the title and abstract of the searched studies using the inclusion and exclusion criteria in order to maintain rigour in this review. If it is unclear from the title and abstract if a study should be included, the full text will be reviewed prior to exclusion. Differences will be discussed by the two reviewers and disagreements will be resolved through discussion with the expert reference

For peer review only - http://bmjopen.bmj.com/site/about/guidelines.xhtml

committee until consensus is reached. This process will facilitate dialogue among reviewers and the expert reference committee in an effort to include all relevant data.

Data extraction and study appraisal

In realist synthesis, data extraction is more akin to note taking.[22] Each document included in the study will be reviewed using a bespoke data extraction form to facilitate and organise note taking (supplementary file 2). The variables extracted will include: 1) title; 2) authors; 3) year of publication; 4) type of document/study 5) countries involved; 6) international/regional organisations involved; 7) specific foodborne hazard; 8) implicated food item; 9) name and details of communication tool used; 10) factors that facilitated the use of the tool; 11) factors that limited the use of the tool; 12) conclusions made by the authors with respect to the use of the tools; 13) recommendations made by the authors with respect to improving international communication during international food safety events; 14) any other contextual factors; 15) any other underlying mechanisms; and 16) Points of discussion to raise with expert reference committee.

As per the RAMESES guidelines, the quality appraisal will be made on the basis of how each study contributes to the development of C-M-O configurations. [21] In a realist synthesis, quality is determined by assessing two criteria: 1) relevance; and 2) rigour.[31] Relevance refers to the degree to which the information in the study fits within the scope of the review and rigor refers to methodological rigor and the degree to which conclusions reached in the study are appropriately drawn based on the research design employed.[22] To aid in this assessment, the Mixed Methods Appraisal Tool[31] will be utilised (see supplementary file 3), but will only be applied to the relevant aspects of each study under review and not necessary the whole study. This tool allows for assessment of multiple studies designs concurrently, it has theoretical and content validity and it has also been tested for efficiency and reliability.[32, 33] The relevance and rigor of each of the included studies will be evaluated by two reviewers who will document a summary of their assessment in tabular format for

For peer review only - http://bmjopen.bmj.com/site/about/guidelines.xhtml

consideration during analysis. Differences will be discussed by the two reviewers and disagreements will be resolved through discussion with the expert reference committee until consensus is reached.

Data synthesis

A thematic approach will be applied in order to record patterns in context, mechanisms and outcomes within each document reviewed and then across documents. These patterns will be compared to the original programme theory to determine if they support, expand or refute its configuration. The approach is abductive, utilising both inductive and deductive analytic processes to understand the C-M-O configurations. As articulated in the RAMESES guidelines, the intention here will be to interrogate the C-M-O configurations and not to provide quantifiable summary data from the studies reviewed.[21] If the reviewed data do not fully explain the initial theory or if new theories emerge through this process, the literature search will be refocused in order to adequately synthesise a final programme theory with supporting thematic explanations.

Validity

Using an iterative approach to understand how different tools facilitate cross-border communication during international food safety events, why they are used, by whom, and for what purpose, will allow researchers to revisit the C-M-O configurations throughout the process as data from the literature is collected.[22] This practice and the intentional inclusion of context in the analysis will improve external validity and the potential generalisability of mechanisms identified in the review.[21] Further, the utilisation of an expert reference committee to elicit feedback, identify additional publications and review the programme theories as they are developed, serves to further bolster internal validity.

Ethics

For peer review only - http://bmjopen.bmj.com/site/about/guidelines.xhtml

Ethical approval is not required for this review as it does not involve primary research. However, it will be conducted according to the appropriate ethical standards of accuracy, utility, usefulness, accountability, feasibility and propriety.[27]

Patient and Public Involvement

Patients were not involved in the design of this study. As described earlier, the first author has involved a group of external experts to develop and refine the initial programme theory presented in this protocol.

Discussion

Significance

Increasingly, globalisation of our food supply necessitates international communication and coordination among food safety and public health professionals in order to prevent, detect, and respond to foodborne disease outbreaks and instances of food contamination that affect more than one country. Rigorous research is needed to understand how the various tools used to facilitate communication are actually working and in what contexts. The knowledge gained from this study will provide valuable lessons on how different tools facilitate cross-border communication during international food safety events, why they are used, by whom, and for what purpose.

Dissemination

The RAMESES publication standards will be followed to report the findings of this review. Upon completion, the final manuscript will be shared with members of INFOSAN, which includes public health and food safety professionals from national government agencies in 188 countries. Further, it is the intent of the author to submit the review for publication in a leading peer-reviewed journal focusing on globalization and health. The review will also be submitted as a chapter in the first author's PhD thesis to be submitted to Lancaster University.

For peer review only - http://bmjopen.bmj.com/site/about/guidelines.xhtml

Limitations

One limitation of this review is that it will only be conducted in English and therefore may introduce an element of language bias. Another limitation relates to challenges in reproducibility of realist reviews compared to traditional systematic reviews because of reliance on experience, prudence and intuition to judge which studies are most relevant to include.[21]

Conclusion

Responding to international food safety events is complex, in part because of the globalised nature of our food supply and the involvement of numerous international stakeholders. In this paper, a protocol for conducting a realist synthesis on different tools to facilitate cross-border communication during international food safety events has been presented which has important but under-studied implications on global efforts to mitigate the burden of foodborne illness resulting from internationally distributed food. The programme theory to be developed will be useful to policy makers and those coordinating the operation of communication tools currently in use, who may adapt components of the tools according to different contextual factors to promote, support and improve their use. By improving international coordination and communication during international food safety events, the global burden of foodborne disease can be mitigated.

Acknowledgements: The authors wish to acknowledge the members of expert review committee for their input during the development of the initial programme theory and for reviewing earlier drafts of this manuscript. Members include: Mr Jan Baele (RASFF Coordinator, European Commission, Belgium), Mr Juan Ortuzar (International Affairs Coordinator, Chilean Agency for Food Safety and Quality, Chile), Dr Caroline Merten (Scientific Officer, Scientific Committee and Emerging Risk Unit, Italy), Ms Jenny Bishop (Team Manager, Food Compliance Services Group, Operations Branch, Ministry for Primary Industries, New Zealand), Dr Jorgen Schlundt (Director, Nanyang Technological University Food Technology Center, Singapore), Dr Peter Ben Embarek (Scientist, INFOSAN Secretariat, Department of Food Safety and Zoonoses, WHO, Switzerland), Mr Adam Bradshaw

For peer review only - http://bmjopen.bmj.com/site/about/guidelines.xhtml

(Technical Officer, INFOSAN Secretariat, Department of Food Safety and Zoonoses, WHO, Switzerland), Mr Raul Garcia (Consultant, INFOSAN Secretariat, Department of Food Safety and Zoonoses, WHO, Switzerland) and Dr Peter Gerner-Smidt (Chief, Enteric Diseases Laboratory Branch, Centers for Disease Control and Prevention, USA).

Authors' contributions: CS conceived the original idea, designed the study, drafted the manuscript and approved the final document. CM drafted the manuscript and approved the final document. CS is a staff member of the World Health Organization. The author alone is responsible for the views expressed in this publication and they do not necessarily represent the views, decisions or policies of the World Health Organization.

Funding statement: This study is funded by the World Health Organization.

Competing interests: None

References

1. World Health Organization. (2015a). *Food safety*. Retrieved from: http://www.who.int/mediacentre/factsheets/fs399/en/

2. World Health Organization. (2015b). *Global burden of foodborne disease report*. Retrieved from: http://apps.who.int/iris/bitstream/10665/200046/1/WHO_FOS_15.02_eng.pdf?ua=1

3. World Health Organization. (2014). *Advancing food safety initiative: strategic plan for food safety including foodborne zoonoses 2013-2022*. Retrieved from: http://www.who.int/foodsafety/publications/strategic-plan/en/

4. World Bank. (2018). *The Safe Food Imperative : Accelerating Progress in Low- and Middle-Income Countries.* Retrieved from: https://openknowledge.worldbank.org/handle/10986/30568

5. World Health Organization. (2018a). *Food safety Data by WHO region*. Retrieved from: http://apps.who.int/gho/data/view.main.IHRREG11v?lang=en

6. Robert Koch Institute. (2011). *Technical Report EHEC/HUS O104:H4 Outbreak.*Germany, May/June 2011. Retrieved from: http://www.rki.de/EN/Home/EHEC_Report.pdf?__blob=publicationFile

7. Bernard, H., Faber, M., Wilking, H., Haller, S., Höhle, M., & Schielke, A. et al. (2014). Large multistate outbreak of norovirus gastroenteritis associated with frozen strawberries, Germany, 2012. *Eurosurveillance*, *19*(8), 20719. http://dx.doi.org/10.2807/1560-7917.es2014.19.8.20719

For peer review only - http://bmjopen.bmj.com/site/about/guidelines.xhtml

8. Gossner, C., Schlundt, J., Ben Embarek, P., Hird, S., Lo-Fo-Wong, D., & Beltran, J. et al. (2009). The Melamine Incident: Implications for International Food and Feed Safety. *Environmental Health Perspectives*, *117*(12), 1803-1808. http://dx.doi.org/10.1289/ehp.0900949

9. Severi, E., Verhoef, L., Thornton, L., Guzman-Herrador, B., Faber, M., & Sundqvist, L. et al. (2015). Large and prolonged food-borne multistate hepatitis A outbreak in Europe associated with consumption of frozen berries, 2013 to 2014. *Eurosurveillance*, *20*(29), 21192. http://dx.doi.org/10.2807/1560-7917.es2015.20.29.21192

10. World Health Organization. (2018b). *Listeriosis – South Africa*. Retrieved from: http://www.who.int/csr/don/28-march-2018-listeriosis-south-africa/en/

11. World Health Organization. (2018c). *INFOSAN Quarterly Summary*. Retrieved from: http://www.who.int/foodsafety/areas_work/infosan/INFOSAN-QS/en/

12. Food and Agriculture Organization of the United Nations. (2015). *The State of Agricultural Commodity Markets.* Retrieved from: http://www.fao.org/3/a-i5090e.pdf

13. International Food Policy Research Institute. (2018). 2018 Global Food Policy Report. Retrieved from: http://ebrary.ifpri.org/utils/getfile/collection/p15738coll2/id/132273/filename/132488.pdf

14. World Health Organization. (2000). *Resolution WHA53.15. Food Safety*. Retrieved from: http://apps.who.int/gb/archive/pdf_files/WHA53/ResWHA53/15.pdf

15. World Health Organization. (2002). *Resolution WHA55.16. Global public health response to natural occurrence, accidental release or deliberate use of biological and chemical agents or radionuclear material that affect health Food Safety*. Retrieved from: http://apps.who.int/gb/archive/pdf_files/WHA55/ewha5516.pdf

16. Food and Agriculture Organization of the United Nations/World Health Organization. (2013). *INFOSAN Activity Report 2011/2012.* Retrieved from: http://www.who.int/foodsafety/publications/activity-report-2012/en/

17. Food and Agriculture Organization of the United Nations/World Health Organization. (2014). *INFOSAN Activity Report 2013*. Retrieved from: http://www.who.int/foodsafety/publications/activity-report-2013/en/

18. Food and Agriculture Organization of the United Nations/World Health Organization. (2016). *INFOSAN Activity Report 2014/2015*. Retrieved from: http://apps.who.int/iris/bitstream/10665/246204/1/9789241510592-eng.pdf

19 Food and Agriculture Organization of the United Nations/World Health Organization. (2018). *INFOSAN Activity Report 2016/2017*. Retrieved from: https://www.who.int/foodsafety/publications/INFOSAN_Activity_Report_2016-17_EN.PDF?ua=1

20. Savelli, C., Bradshaw, A., Ben Embarek, P., Mateus, C. (in press). The FAO/WHO International Food Safety Authorities Network (INFOSAN) in Review, 2004-2018: Learning from the past and looking to the future. *Foodborne Pathogens and Disease.*

For peer review only - http://bmjopen.bmj.com/site/about/guidelines.xhtml

21. Wong, G., Greenhalgh, T., Westhorp, G., Buckingham, J., & Pawson, R. (2013). RAMESES publication standards: realist syntheses. *BMC Medicine*, *11*(1). http://dx.doi.org/10.1186/1741-7015-11-21

22. Pawson, R., Greenhalgh, T., Harvey, G., & Walshe, K. (2005). Realist review - a new method of systematic review designed for complex policy interventions. *Journal Of Health Services Research & Policy*, *10*(1_suppl), 21-34. http://dx.doi.org/10.1258/1355819054308530

23. Giddens, A. (1990). *The consequences of modernity* (1st ed.). Stanford, Calif.: Stanford University Press.

24. Giddens, A. (1991). *Modernity and self-identity*. (1st ed). Stanford, Calif.: Stanford University Press.

25. Robinson, W. (2011). Globalization and the sociology of Immanuel Wallerstein: A critical appraisal. *International Sociology*, *26*(6), 723-745. http://dx.doi.org/10.1177/0268580910393372

26. Gunderson, L. and Holling, C. (2002). *Panarchy*. (1st ed). Washington, DC: Island Press.

27. Mertens, D. (2009). *Transformative research and evaluation*. 1st ed. New York: Guilford Press.

28. Thompson, S., Clark, A., Molzahn, A., Klarenbach, S., & Tonelli, M. (2016). Increasing the uptake of exercise programs in the dialysis unit: a protocol for a realist synthesis. *Systematic Reviews*, *5*(1). http://dx.doi.org/10.1186/s13643-016-0224-6

29. Pitzul, K., Lane, N., Voruganti, T., Khan, A., Innis, J., Wodchis, W., & Baker, G. (2015). Role of context in care transition interventions for medically complex older adults: a realist synthesis protocol. *BMJ Open*, *5*(11), e008686. http://dx.doi.org/10.1136/bmjopen-2015-008686

30. Durham, J. & Blondell, S. (2014). Research protocol: a realist synthesis of cross-border patient mobility from low-income and middle-income countries. *BMJ Open*, *4*(11), e006514. http://dx.doi.org/10.1136/bmjopen-2014-006514

31. Pawson, R. (2006). *Evidence based policy: a realist perspective*. London: Sage.

32. Pluye, P., Gagnon, M., Griffiths, F., & Johnson-Lafleur, J. (2009). A scoring system for appraising mixed methods research, and concomitantly appraising qualitative, quantitative and mixed methods primary studies in Mixed Studies Reviews. *International Journal Of Nursing Studies*, *46*(4), 529-546. http://dx.doi.org/10.1016/j.ijnurstu.2009.01.009

33. Pace, R., Pluye, P., Bartlett, G., Macaulay, A., Salsberg, J., Jagosh, J., & Seller, R. (2012). Testing the reliability and efficiency of the pilot Mixed Methods Appraisal Tool (MMAT) for systematic mixed studies review. *International Journal Of Nursing Studies*, *49*(1), 47-53. http://dx.doi.org/10.1016/j.ijnurstu.2011.07.002

For peer review only

For peer review only - http://bmjopen.bmj.com/site/about/guidelines.xhtml

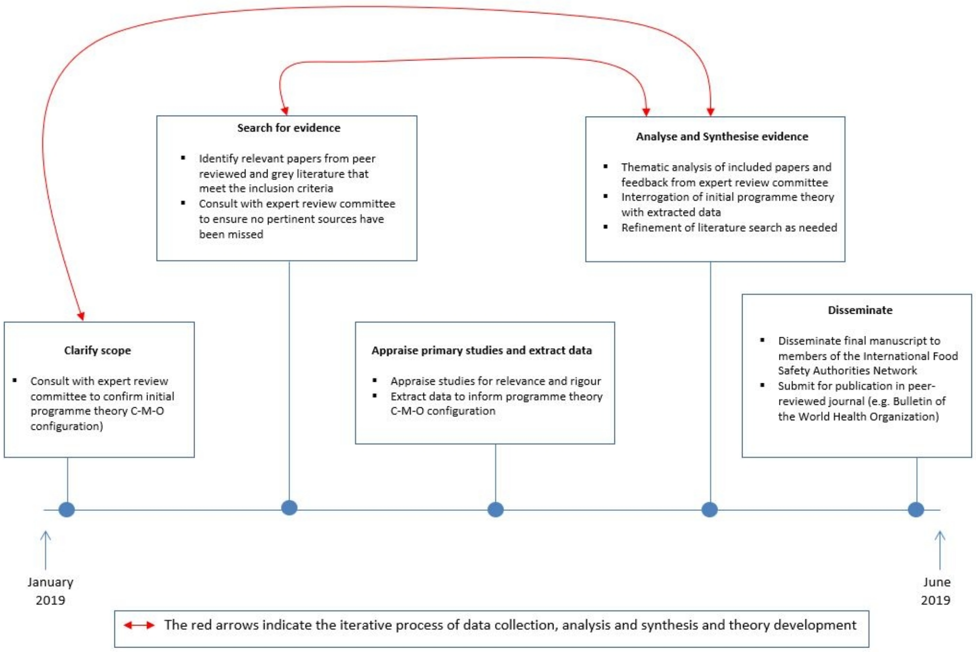

Overview of the stages of this review.

275x207mm (96 x 96 DPI)

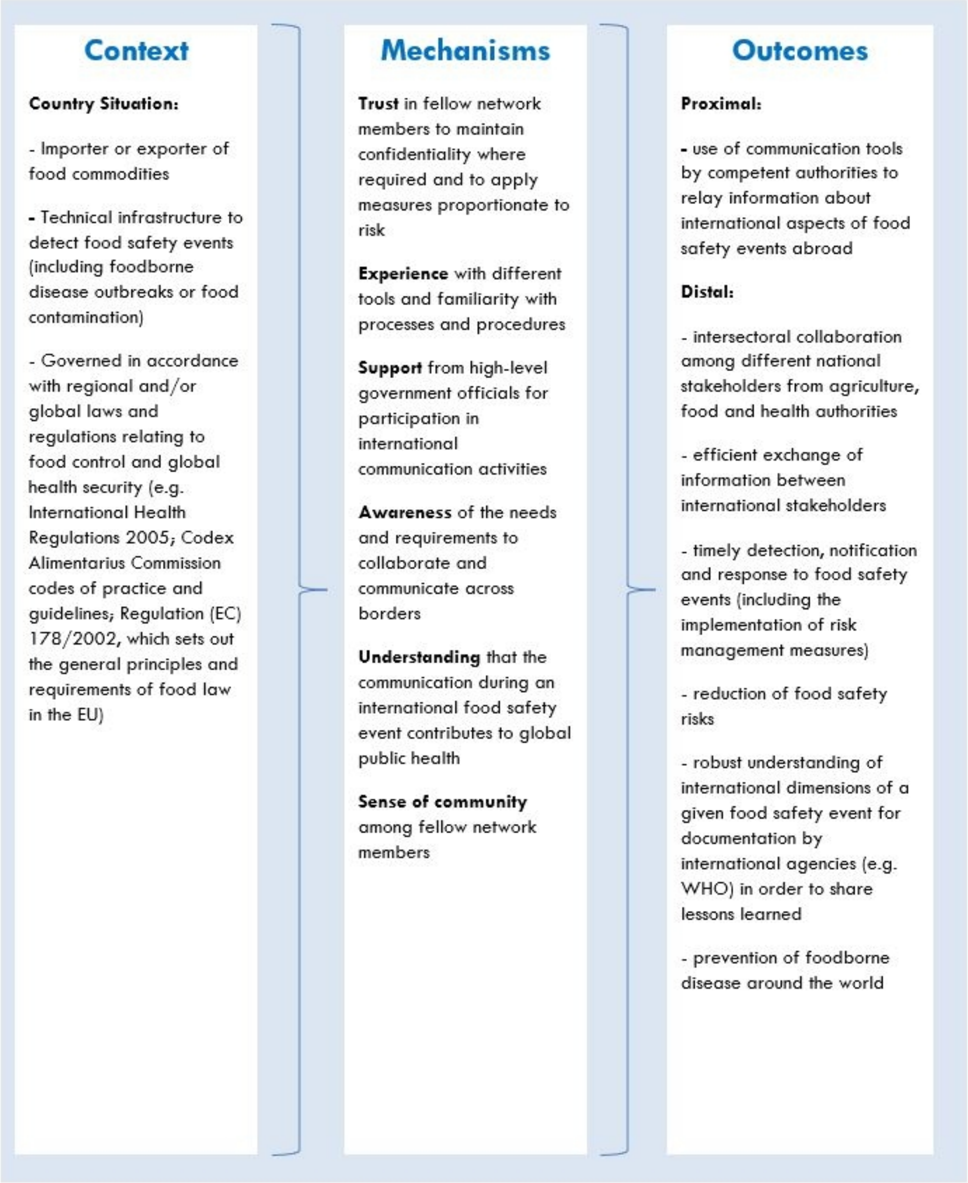

Overview of the initial programme theory C-M-O configuration.

186x226mm (96 x 96 DPI)

Supplementary File 1 – Preliminary inventory of communication tools currently used to exchange information during international food safety events*

Name of tool	Description	Type of Users	Primary Mode of Communication	Primary type of information exchanged	Coordinating Responsibly
International Food Safety Authorities Network<br><br>(INFOSAN)	Rapid sharing of information during food safety emergencies to stop the spread of contaminated food from one country to another; facilitates the sharing of experiences and tested solutions in and between countries in order to optimise future interventions to protect the health of consumers; launch in 2004	Officially designated government officials from multiple sectors involved in food safety (e.g. health, agriculture, veterinary services, trade, etc.); Members from 186 countries + European Commission (European Centre for Disease Control (ECDC), European Food Safety Authority (EFSA), Rapid Alert System for Food and Feed (RASFF) Commission Contact Point)	Web-based communication platform<br><br>(INFOSAN Community Website, members only)	Food contamination information along with export details; foodborne outbreak information including related epidemiologic, laboratory and traceability information	Secretariat at WHO; (Jointly managed by FAO and WHO)
For more information, see: http://www.who.int/foodsafety/areas_work/infosan/en/					
Rapid Alert System for Food and Feed<br><br>(RASFF)	Provides food and feed control authorities with an effective tool to exchange information about measures taken responding to serious risks detected in relation to food or feed; launched in 1979	Members from 28 national food safety authorities in the EU + EFSA, ESA, Norway, Liechtenstein, Iceland and Switzerland & WHO	Web-based communication platforms (RASFF Window, members only)	Food contamination information along with export details; foodborne outbreak information including related epidemiologic, laboratory and traceability information	Directorate General for Health and Food Safety<br><br>(DG SANTE, European Commission)
For more information, see: https://ec.europa.eu/food/safety/rasff_en					
Early Warning and Response system of the European Union<br><br>(EWRS)	Confidential computer system allowing Member States to send alerts about events with a potential impact on the EU, share information, and coordinate their response	Members from 28 Public Health Authorities of the EU Member States, which have been designated officially by the Government of their Country as members of the EWRS, and the competent service of the European Commission & WHO	Web-based communication platform (members only)	Foodborne disease surveillance information	ECDC<br><br>(European Commission)
For more information, see: http://www.eurosurveillance.org/ViewArticle.aspx?ArticleId=666					
Epidemic Intelligence Information System for Foodborne and Waterborne diseases and Zoonoses<br><br>(EPIS-FWD)	Facilitates the early detection and assessment of multi-country/multinational molecular typing clusters and outbreaks of Foodborne and Waterborne diseases and Zoonoses	The platform connects epidemiologists and microbiologists from 52 countries: 28 EU Member States, three countries of the European Economic Area (EEA) - Iceland, Norway and Liechtenstein - and 21 other non-EU countries & WHO	Web-based communication platform (members only)	Molecular typing clusters and foodborne, waterborne and Zoonotic outbreak details;	ECDC (European Commission)
For more information, see: http://ecdc.europa.eu/en/aboutus/what-we-do/epidemic-intelligence/Pages/EpidemicIntelligence_Tools.aspx					
Hepatitis A Lab-Network	Global network of hepatitis A reference laboratories	Scientists working in hepatitis A reference laboratories	Email; Web-based electronic database (members only)	Molecular and epidemiological data on hepatitis A virus	National Institute for Public Health and the Environment

(HAVNet)					(RIVM, The Netherlands)
For more information, see: http://www.eurosurveillance.org/ViewArticle.aspx?ArticleId=21356					
PulseNet International	Network of national and regional laboratory networks dedicated to tracking foodborne infections world-wide; Each laboratory utilises standardised genotyping methods, sharing information in real-time	Scientists working in public health laboratories and academic and medical institutions, including 10 countries in the Eastern Mediterranean, 16 countries in the Americas, 11 countries in the Western Pacific, 11 countries in Africa, 31 countries in Europe	PulseNet International forum hosted by PulseNet Canada (Web-based, members only, communication platform)	Genotyping information from foodborne pathogens (some genomic data)	US Centers for Disease Control (CDC)
For more information, see: http://www.pulsenetinternational.org/					
ASEAN Rapid Alert System for Food and Feed	Regional network modelled after the European RASFF system involving 10 ASEAN Member States.	Competent Authorities in Food Safety and Public Health of all ASEAN Member States (10 countries)	web-based application	Information on direct or indirect risks to human deriving from food or feed being traded in ASEAN and measures taken to prevent them entering the food chain	National Bureau of Agricultural Commodity and Food Standards (ACFS),<br><br>Ministry of Agriculture and Cooperatives, Thailand.
For more information, see: http://arasff.net/index.php					
Bi-lateral agreements	Many national authorities have established bi-lateral agreements with foreign agencies for the purpose of exchanging information during international food safety events.	National authorities.	Email or telephone.	Could be any type of information required to respond to an international food safety event.	National authorities.

*This list has been informed by the author's experience as the WHO secretariat of INFOSAN, the results from a scoping search, and input from the Expert Reference Committee

For peer review only

Supplemental File 2 – Detailed Search Strategies for Web of Science, Embase, MEDLINE, PubMed and CINAHL

Web of Science

Search History:

Set	Results	Save History / Create Alert Open Saved History
# 7	1,027	#6 AND #5 AND #4<br>*Indexes=SCI-EXPANDED, SSCI, A&HCI, CPCI-S, CPCI-SSH, ESCI Timespan=1995-2019*
# 6	9,703,534	TS=(systems OR network OR tool OR communication OR notification OR “information exchange”)<br>*Indexes=SCI-EXPANDED, SSCI, A&HCI, CPCI-S, CPCI-SSH, ESCI Timespan=1995-2019*
# 5	806,837	TS=(international OR multi-state OR multi-country OR imported OR exported)<br>*Indexes=SCI-EXPANDED, SSCI, A&HCI, CPCI-S, CPCI-SSH, ESCI Timespan=1995-2019*
# 4	32,199	#3 OR #2 OR #1<br>*Indexes=SCI-EXPANDED, SSCI, A&HCI, CPCI-S, CPCI-SSH, ESCI Timespan=1995-2019*
# 3	26,514	TS=("food safety" OR "food contamination" OR "foodborne diseases")<br>*Indexes=SCI-EXPANDED, SSCI, A&HCI, CPCI-S, CPCI-SSH, ESCI Timespan=1995-2019*
# 2	1,793	TS=(gastroenteritis NEAR/5 (incident* OR emergenc* OR outbreak*))<br>*Indexes=SCI-EXPANDED, SSCI, A&HCI, CPCI-S, CPCI-SSH, ESCI Timespan=1995-2019*
# 1	5,547	TS=(food* NEAR/5 (incident* Or emergenc* OR outbreak*))<br>*Indexes=SCI-EXPANDED, SSCI, A&HCI, CPCI-S, CPCI-SSH, ESCI Timespan=1995-2019*

Embase and MEDLINE

# ▲	Searches	Results
1	(food* adj5 incident*).af.	983
2	(food* adj5 emergenc*).af.	5617
3	(food* adj5 outbreak*).af.	8825
4	(gastroenteritis adj5 incident*).af.	54
5	(gastroenteritis adj5 emergenc*).af.	1206
6	(gastroenteritis adj5 outbreak*).af.	4424
7	("food safety" or "food contamination" or "foodborne diseases").af.	156116
8	(international or multi-state or multi-country or imported or exported).af.	3541525
9	(systems or network or tool or communication or notification or information-exchange).af.	4810679
10	1 or 2 or 3	15272
11	4 or 5 or 6	5640
12	7 or 10 or 11	170450
13	8 and 9 and 12	2835
14	limit 13 to yr="1995 -Current"	2779

PubMed

Search	Add to builder	Query	Items found	Time
#7	Add	Search ((((systems OR network OR tool OR communication OR notification OR "information exchange"))) AND ((international OR multi-state OR multi-country OR imported OR exported))) AND ((((("food safety" OR "food contamination" OR "foodborne diseases"))) OR ((gastroenteritis AND (incident* OR emergenc* OR outbreak*)))) OR ((food* AND (incident* Or emergenc* OR outbreak*))))	1062	10:27:02
#6	Add	Search (systems OR network OR tool OR communication OR notification OR "information exchange")	2269385	10:26:36
#5	Add	Search (international OR multi-state OR multi-country OR imported OR exported)	528235	10:26:03
#4	Add	Search (((("food safety" OR "food contamination" OR "foodborne diseases"))) OR ((gastroenteritis AND (incident* OR emergenc* OR outbreak*)))) OR ((food* AND (incident* Or emergenc* OR outbreak*)))	105011	10:25:24
#3	Add	Search ("food safety" OR "food contamination" OR "foodborne diseases")	74378	10:24:58
#2	Add	Search (gastroenteritis AND (incident* OR emergenc* OR outbreak*))	13879	10:24:38
#1	Add	Search (food* AND (incident* Or emergenc* OR outbreak*))	26356	10:24:21

CINAHL

Search ID#	Search Terms	Search Options	Actions
S7	S4 AND S5 AND S6	Search modes - Find all my search terms	View Results (79) View Details Edit
S6	systems OR network OR tool OR communication OR notification OR "information exchange"	Search modes - Find all my search terms	View Results (836,810) View Details Edit
S5	international OR multi-state OR multi-country OR imported OR exported	Search modes - Find all my search terms	View Results (125,224) View Details Edit
S4	S1 OR S2 OR S3	Search modes - Find all my search terms	View Results (6,148) View Details Edit
S3	"food safety" OR "food contamination" OR "foodborne diseases"	Search modes - Find all my search terms	View Results (5,155) View Details Edit
S2	gastroenteritis N5 (incident* OR emergenc* OR outbreak*)	Search modes - Find all my search terms	View Results (417) View Details Edit
S1	food* N5 (incident* Or emergenc* OR outbreak*)	Search modes - Find all my search terms	View Results (889) View Details Edit

Supplemental File 3 - Data Extraction Form

**EndNote Reference Number**
**Title**
**Authors**
**Year of publication**
**Type of document/study**
**Countries involved (specify where food was produced, where food was distributed, and where illnesses occurred)**
**International/regional organizations involved (specify who took a coordinating role)**
**Specific foodborne hazard**
**Implicated food item (specify how food was implicated, e.g. epidemiologic evidence, laboratory evidence, traceability evidence)**
**Name and details of communication tool(s) used (including who used the tool and what kind of information was exchanged)**
**Factors that facilitated the use of the tools (context or mechanisms)**
**Factors that limited the use of the tool (context or mechanisms)**
**Conclusions made by the authors with respect to the use of the tools (related to context, mechanisms or outcomes)**
**Recommendations made by the authors with respect to improving international communication during international food safety events**
**Any other contextual factors**

For peer review only

**Any other underlying mechanisms**
**Points of discussion to raise with Expert Reference Committee**

For peer review only

Supplemental File 4 - Mixed Methods Appraisal Tool

Pluye, P., Gagnon, M., Griffiths, F., & Johnson-Lafleur, J. (2009). A scoring system for appraising mixed methods research, and concomitantly appraising qualitative, quantitative and mixed methods primary studies in Mixed Studies Reviews. *International Journal Of Nursing Studies*, *46*(4), 529-546. http://dx.doi.org/10.1016/j.ijnurstu.2009.01.009 (updated 2011 version available online: http://mixedmethodsappraisaltoolpublic.pbworks.com/w/file/fetch/84371689/MMAT%202011%20criteria%20and%20tutorial%202011-06-29updated2014.08.21.pdf

PART I. MMAT criteria & one-page template (to be included in appraisal forms)

Types of mixed methods study components or primary studies	Methodological quality criteria (see tutorial for definitions and examples)	Responses			
		Yes	No	Can't tell	Comments
**Screening questions (for all types)**	• Are there clear qualitative and quantitative research questions (or objectives*), or a clear mixed methods question (or objective*)?				
	• Do the collected data allow address the research question (objective)? E.g., consider whether the follow-up period is long enough for the outcome to occur (for longitudinal studies or study components).				
	Further appraisal may be not feasible or appropriate when the answer is 'No' or 'Can't tell' to one or both screening questions.				
**1. Qualitative**	1.1. Are the sources of qualitative data (archives, documents, informants, observations) relevant to address the research question (objective)?				
	1.2. Is the process for analyzing qualitative data relevant to address the research question (objective)?				
	1.3. Is appropriate consideration given to how findings relate to the context, e.g., the setting, in which the data were collected?				
	1.4. Is appropriate consideration given to how findings relate to researchers' influence, e.g., through their interactions with participants?				
**2. Quantitative randomized controlled (trials)**	2.1. Is there a clear description of the randomization (or an appropriate sequence generation)?				
	2.2. Is there a clear description of the allocation concealment (or blinding when applicable)?				
	2.3. Are there complete outcome data (80% or above)?				
	2.4. Is there low withdrawal/drop-out (below 20%)?				
**3. Quantitative non-randomized**	3.1. Are participants (organizations) recruited in a way that minimizes selection bias?				
	3.2. Are measurements appropriate (clear origin, or validity known, or standard instrument; and absence of contamination between groups when appropriate) regarding the exposure/intervention and outcomes?				
	3.3. In the groups being compared (exposed vs. non-exposed; with intervention vs. without; cases vs. controls), are the participants comparable, or do researchers take into account (control for) the difference between these groups?				
	3.4. Are there complete outcome data (80% or above), and, when applicable, an acceptable response rate (60% or above), or an acceptable follow-up rate for cohort studies (depending on the duration of follow-up)?				
**4. Quantitative descriptive**	4.1. Is the sampling strategy relevant to address the quantitative research question (quantitative aspect of the mixed methods question)?				
	4.2. Is the sample representative of the population understudy?				
	4.3. Are measurements appropriate (clear origin, or validity known, or standard instrument)?				
	4.4. Is there an acceptable response rate (60% or above)?				
**5. Mixed methods**	5.1. Is the mixed methods research design relevant to address the qualitative and quantitative research questions (or objectives), or the qualitative and quantitative aspects of the mixed methods question (or objective)?				
	5.2. Is the integration of qualitative and quantitative data (or results*) relevant to address the research question (objective)?				
	5.3. Is appropriate consideration given to the limitations associated with this integration, e.g., the divergence of qualitative and quantitative data (or results*) in a triangulation design?				
	Criteria for the qualitative component (1.1 to 1.4), and appropriate criteria for the quantitative component (2.1 to 2.4, or 3.1 to 3.4, or 4.1 to 4.4), must be also applied.				

*These two items are not considered as double-barreled items since in mixed methods research, (1) there may be research questions (quantitative research) or research objectives (qualitative research), and (2) data may be integrated, and/or qualitative findings and quantitative results can be integrated.

PART II. MMAT tutorial

Types of mixed methods study components or primary studies	Methodological quality criteria
**1. Qualitative**<br><br>Common types of qualitative research methodology include:<br><br>A. Ethnography<br>The aim of the study is to describe and interpret the shared cultural behaviour of a group of individuals.<br><br>B. Phenomenology<br>The study focuses on the subjective experiences and interpretations of a phenomenon encountered by individuals.<br><br>C. Narrative<br>The study analyzes life experiences of an individual or a group.<br><br>D. Grounded theory<br>Generation of theory from data in the process of conducting research (data collection occurs first).<br><br>E. Case study<br>In-depth exploration and/or explanation of issues intrinsic to a particular case. A case can be anything from a decision-making process, to a person, an organization, or a country.<br><br>F. Qualitative description<br>There is no specific methodology, but a qualitative data collection and analysis, e.g., in-depth interviews or focus groups, and hybrid thematic analysis (inductive and deductive).<br><br>Key references: Creswell, 1998; Schwandt, 2001; Sandelowski, 2010.	**1.1. Are the sources of qualitative data (archives, documents, informants, observations) relevant to address the research question (objective)?**<br><br>E.g., consider whether (a) the selection of the participants is clear, and appropriate to collect relevant and rich data; and (b) reasons why certain potential participants chose not to participate are explained.
	**1.2. Is the process for analyzing qualitative data relevant to address the research question (objective)?**<br><br>E.g., consider whether (a) the method of data collection is clear (in depth interviews and/or group interviews, and/or observations and/or documentary sources); (b) the form of the data is clear (tape recording, video material, and/or field notes for instance); (c) changes are explained when methods are altered during the study; and (d) the qualitative data analysis addresses the question.
	**1.3. Is appropriate consideration given to how findings relate to the context, e.g., the setting, in which the data were collected?***<br><br>E.g., consider whether the study context and how findings relate to the context or characteristics of the context are explained (how findings are influenced by or influence the context). "For example, a researcher wishing to observe care in an acute hospital around the clock may not be able to study more than one hospital. (…) Here, it is essential to take care to describe the context and particulars of the case [the hospital] and to flag up for the reader the similarities and differences between the case and other settings of the same type" (Mays & Pope, 1995).<br><br>The notion of context may be conceived in different ways depending on the approach (methodology) tradition.
	**1.4. Is appropriate consideration given to how findings relate to researchers' influence, e.g., through their interactions with participants?***<br><br>E.g., consider whether (a) researchers critically explain how findings relate to their perspective, role, and interactions with participants (how the research process is influenced by or influences the researcher); (b) researcher's role is influential at all stages (formulation of a research question, data collection, data analysis and interpretation of findings); and (c) researchers explain their reaction to critical events that occurred during the study.<br><br>The notion of reflexivity may be conceived in different ways depending on the approach (methodology) tradition. E.g., "at a minimum, researchers employing a generic approach [qualitative description] must explicitly identify their disciplinary affiliation, what brought them to the question, and the assumptions they make about the topic of interest" (Caelli, Ray & Mill, 2003, p. 5).

*See suggestion on the MMAT wiki homepage (under '2011 version'): Independent reviewers can establish a common understanding of these two items prior to beginning the critical appraisal.

Types of mixed methods study components or primary studies	Methodological quality criteria
**2. Quantitative randomized controlled (trials)**<br><br>Randomized controlled clinical trial: A clinical study in which individual participants are allocated to intervention or control groups by randomization (intervention assigned by researchers).<br><br>Key references: Higgins & Green, 2008; Porta, 2008; Oxford Center for Evidence based medicine, 2009.	**2.1. Is there a clear description of the randomization (or an appropriate sequence generation)?**<br><br>In a randomized controlled trial, the allocation of a participant (or a data collection unit, e.g., a school) into the intervention or control group is based solely on chance, and researchers describe how the randomization schedule is generated. "A simple statement such as 'we randomly allocated' or 'using a randomized design' is insufficient".<br><br>*Simple randomization:* Allocation of participants to groups by chance by following a predetermined plan/sequence. "Usually it is achieved by referring to a published list of random numbers, or to a list of random assignments generated by a computer".<br><br>*Sequence generation:* "The rule for allocating interventions to participants must be specified, based on some chance (random) process". Researchers provide sufficient detail to allow a readers' appraisal of whether it produces comparable groups. E.g., blocked randomization (to ensure particular allocation ratios to the intervention groups), or stratified randomization (randomization performed separately within strata), or minimization (to make small groups closely similar with respect to several characteristics).
	**2.2. Is there a clear description of the allocation concealment (or blinding when applicable)?**<br><br>*The allocation concealment protects assignment sequence until allocation.* E.g., researchers and participants are unaware of the assignment sequence up to the point of allocation. E.g., group assignment is concealed in opaque envelops until allocation.<br><br>*The blinding protects assignment sequence after allocation.* E.g., researchers and/or participants are unaware of the group a participant is allocated to during the course of the study.
	**2.3. Are there complete outcome data (80% or above)?**<br><br>E.g., almost all the participants contributed to almost all measures.
	**2.4. Is there low withdrawal/drop-out (below 20%)?**<br><br>E.g., almost all the participants completed the study.

Types of mixed methods study components or primary studies	Methodological quality criteria
**3. Quantitative non-randomized**<br><br>Common types of design include (A) non-randomized controlled trials, and (B-C-D) observational analytic study or component where the intervention/exposure is defined/assessed, but not assigned by researchers.<br><br>A. Non-randomized controlled trials<br>The intervention is assigned by researchers, but there is no randomization, e.g., a pseudo-randomization. A non-random method of allocation is not reliable in producing alone similar groups.<br><br>B. Cohort study<br>Subsets of a defined population are assessed as exposed, not exposed, or exposed at different degrees to factors of interest. Participants are followed over time to determine if an outcome occurs (prospective longitudinal).<br><br>C. Case-control study<br>Cases, e.g., patients, associated with a certain outcome are selected, alongside a corresponding group of controls. Data is collected on whether cases and controls were exposed to the factor under study (retrospective).<br><br>D. Cross-sectional analytic study<br>At one particular time, the relationship between health-related characteristics (outcome) and other factors (intervention/exposure) is examined. E.g., the frequency of outcomes is compared in different population sub-groups according to the presence/absence (or level) of the intervention/exposure.<br><br>Key references for observational analytic studies: Higgins & Green, 2008; Wells, Shea, O'Connell, Peterson, et al., 2009.	**3.1. Are participants (organizations) recruited in a way that minimizes selection bias?**<br><br>At recruitment stage:<br><br>For cohort studies, e.g., consider whether the exposed (or with intervention) and non-exposed (or without intervention) groups are recruited from the same population.<br>For case-control studies, e.g., consider whether same inclusion and exclusion criteria were applied to cases and controls, and whether recruitment was done independently of the intervention or exposure status.<br>For cross-sectional analytic studies, e.g., consider whether the sample is representative of the population.
	**3.2. Are measurements appropriate (clear origin, or validity known, or standard instrument; and absence of contamination between groups when appropriate) regarding the exposure/intervention and outcomes?**<br><br>At data collection stage:<br><br>E.g., consider whether (a) the variables are clearly defined and accurately measured; (b) the measurements are justified and appropriate for answering the research question; and (c) the measurements reflect what they are supposed to measure.<br><br>For non-randomized controlled trials, the intervention is assigned by researchers, and so consider whether there was absence/presence of a contamination. E.g., the control group may be indirectly exposed to the intervention through family or community relationships.
	**3.3. In the groups being compared (exposed vs. non-exposed; with intervention vs. without; cases vs. controls), are the participants comparable, or do researchers take into account (control for) the difference between these groups?**<br><br>At data analysis stage:<br><br>For cohort, case-control and cross-sectional, e.g., consider whether (a) the most important factors are taken into account in the analysis; (b) a table lists key demographic information comparing both groups, and there are no obvious dissimilarities between groups that may account for any differences in outcomes, or dissimilarities are taken into account in the analysis.
	**3.4. Are there complete outcome data (80% or above), and, when applicable, an acceptable response rate (60% or above), or an acceptable follow-up rate for cohort studies (depending on the duration of follow-up)?**

Types of mixed methods study components or primary studies	Methodological quality criteria
**4. Quantitative descriptive studies**<br><br>Common types of design include single-group studies:<br><br>A. Incidence or prevalence study without comparison group<br>In a defined population at one particular time, what is happening in a population, e.g., frequencies of factors (importance of problems), is described (portrayed).<br><br>B. Case series<br>A collection of individuals with similar characteristics are used to describe an outcome.<br><br>C. Case report<br>An individual or a group with a unique/unusual outcome is described in details.<br><br>Key references: Critical Appraisal Skills Programme, 2009; Draugalis, Coons & Plaza, 2008.	**4.1. Is the sampling strategy relevant to address the quantitative research question (quantitative aspect of the mixed methods question)?**<br><br>E.g., consider whether (a) the source of sample is relevant to the population under study; (b) when appropriate, there is a standard procedure for sampling, and the sample size is justified (using power calculation for instance).
	**4.2. Is the sample representative of the population understudy?**<br><br>E.g., consider whether (a) inclusion and exclusion criteria are explained; and (b) reasons why certain eligible individuals chose not to participate are explained.
	**4.3. Are measurements appropriate (clear origin, or validity known, or standard instrument)?**<br><br>E.g., consider whether (a) the variables are clearly defined and accurately measured; (b) measurements are justified and appropriate for answering the research question; and (c) the measurements reflect what they are supposed to measure.
	**4.4. Is there an acceptable response rate (60% or above)?**<br><br>The response rate is not pertinent for case series and case report. E.g., there is no expectation that a case series would include all patients in a similar situation.

Types of mixed methods study components or primary studies	Methodological quality criteria
**5. Mixed methods**<br><br>Common types of design include:<br><br>A. Sequential explanatory design<br>The quantitative component is followed by the qualitative. The purpose is to explain quantitative results using qualitative findings. E.g., the quantitative results guide the selection of qualitative data sources and data collection, and the qualitative findings contribute to the interpretation of quantitative results.<br><br>B. Sequential exploratory design<br>The qualitative component is followed by the quantitative. The purpose is to explore, develop and test an instrument (or taxonomy), or a conceptual framework (or theoretical model). E.g., the qualitative findings inform the quantitative data collection, and the quantitative results allow a generalization of the qualitative findings.<br><br>C. Triangulation design<br>The qualitative and quantitative components are concomitant. The purpose is to examine the same phenomenon by interpreting qualitative and quantitative results (bringing data analysis together at the interpretation stage), or by integrating qualitative and quantitative datasets (e.g., data on same cases), or by transforming data (e.g., quantization of qualitative data).<br><br>D. Embedded design<br>The qualitative and quantitative components are concomitant. The purpose is to support a qualitative study with a quantitative sub-study (measures), or to better understand a specific issue of a quantitative study using a qualitative sub-study, e.g., the efficacy or the implementation of an intervention based on the views of participants.<br><br>Key references: Creswell & Plano Clark, 2007; O'Cathain, 2010.	**5.1. Is the mixed methods research design relevant to address the qualitative and quantitative research questions (or objectives), or the qualitative and quantitative aspects of the mixed methods question (or objective)?**<br><br>E.g., the rationale for integrating qualitative and quantitative methods to answer the research question is explained.<br><br>**5.2. Is the integration of qualitative and quantitative data (or results) relevant to address the research question (objective)?**<br><br>E.g., there is evidence that data gathered by both research methods was brought together to form a complete picture, and answer the research question; authors explain when integration occurred (during the data collection-analysis or/and during the interpretation of qualitative and quantitative results); they explain how integration occurred and who participated in this integration.<br><br>**5.3. Is appropriate consideration given to the limitations associated with this integration, e.g., the divergence of qualitative and quantitative data (or results)?**

review only

Utilisation of tools to facilitate cross-border communication during international food safety events, 1995-2019: A realist synthesis protocol

Journal:	*BMJ Open*
Manuscript ID	bmjopen-2019-030593.R1
Article Type:	Protocol
Date Submitted by the Author:	15-Jul-2019
Complete List of Authors:	Savelli, Carmen; Organisation mondiale de la Sante, Food Safety and Zoonoses; Lancaster University Faculty of Health and Medicine, Health Research<br>Mateus, Ceu; University of Lancaster, Division of Health Research
**Primary Subject Heading**:	Global health
Secondary Subject Heading:	Global health, Public health
Keywords:	Realist review, contextual factors, food safety, foodborne disease outbreaks, international networks, communication tools

SCHOLARONE™ Manuscripts

Title: Utilisation of tools to facilitate cross-border communication during international food safety events, 1995-2019: A realist synthesis protocol

Authors: Carmen Joseph Savelli[1,2], Céu Mateus[2]

1) World Health Organization, Geneva, Switzerland
2) Lancaster University, Lancaster, United Kingdom

Corresponding Author: Carmen Joseph Savelli
World Health Organization
Food Safety and Zoonoses
Avenue Appia 20
1211, Geneva, Switzerland
savellic@who.int
+41 22 79 13234

Key words: realist review – contextual factors – food safety – foodborne disease outbreaks – international networks – communication tools

Word Count: 5187

For peer review only

For peer review only - http://bmjopen.bmj.com/site/about/guidelines.xhtml

Abstract

Introduction: Efficient communication and coordination between countries is needed for prevention, detection and response to international food safety events. While communication tools exist, current evidence suggests that they are only effective within certain contexts and only cover certain geographic areas. There is a need to unpack and explore the mechanisms of how and in what context such communication tools and their components are effective at facilitating international communication and coordination to keep food safe and mitigate the burden of foodborne disease around the globe.

Methods and analysis: A realist synthesis will be undertaken to understand how and why certain processes and structures of communication tools, utilised during international food safety events, influence their utility and effectiveness according to different contextual factors. The focus of this review is explanatory and aims to develop and refine theory regarding how contextual factors trigger specific processes and mechanisms to produce outcomes. Using the realist context-mechanism-outcome configuration of theory development, a range of sources have been utilised to develop the initial programme theory, including the author's experience, a scoping review of published papers and grey literature, and input from an expert reference committee. To support, expand or refute the initial theory, data will be synthesised from published literature and input from the expert reference committee.

Ethics and dissemination: Ethical approval is not required for this review as it does not involve primary research. However, it will be conducted according to the appropriate ethical standards of accuracy, utility, usefulness, accountability, feasibility and propriety. The RAMESES publication standards will be followed to report the findings of this review. Upon completion, the final manuscript will be shared with members of the FAO/WHO International Food Safety Authorities Network (INFOSAN) and published in a peer reviewed journal.

Strengths and limitations of the study:

For peer review only

For peer review only - http://bmjopen.bmj.com/site/about/guidelines.xhtml

- Uses realist methods to explore contextual factors and underpinning causal mechanisms of complex international communication during food safety events.
- Utilization of an international expert review committee consisting of national government officials, international civil servants and academics to ensure complete coverage of the literature.
- Formulation of the context-mechanism-outcome programme theory relies heavily on published literature and therefore may be subject to publication bias.
- Review findings will be context-specific and therefore must be considered within the context of this research.

Systematic review registration: This protocol has been registered with PROSPERO, registration number: <<to be added>>

Background

Access to sufficient amounts of safe and nutritious food is a basic requirement for human health. However, around the world unsafe food is known to cause more than 200 acute and chronic diseases, ranging from diarrhoea to cancer.[1] In 2015, the first estimates of the global burden of foodborne diseases were reported by the WHO, indicating that 31 hazards (including bacteria, viruses, parasites, toxins and chemicals) were responsible for 600-million cases of foodborne diseases and 420,000 deaths worldwide in 2010.[2] This burden was disproportionately felt by children under five years of age who accounted for 40% of foodborne disease cases and 125,000 deaths. [2] While foodborne diseases are observed worldwide, Africa, South-East Asia and the Eastern Mediterranean regions report the highest burden.[2] In such high-burden areas, unsafe food presents additional consequences beyond disease burden, impeding socioeconomic development, overloading strained healthcare systems and damaging national economies, trade and tourism.[3] Furthermore, a 2018 study by the World Bank[4] indicates that unsafe food costs low- and middle-income economies approximately $100 billion USD in lost productivity and medical expenses each year.

For peer review only

For peer review only - http://bmjopen.bmj.com/site/about/guidelines.xhtml

Foodborne diseases are preventable, however, prevention requires investment and coordinated action across multiple sectors to strengthen national food safety systems. Multiple agencies responsible for health, agriculture, veterinary services, trade, and several others must work together to build a strong and resilient national food safety system. The WHO has identified several core capacities that national governments should develop to safeguard national food supplies. The development of such core capacities is evaluated yearly by the WHO to determine whether countries have established functional mechanisms for the detection, prevention and response to foodborne disease and food contamination events. Data from 2017 indicate that 78% of the attributes of core capacities required for food safety have been developed globally, although disparities exist between regions. For example, 90% of the required core capacities have been achieved in Europe, while in Africa, only 54% of the core capacities have been achieved.[5]

An international food safety event results when unsafe food produced in one country, is exported to at least one other country. Recent international food safety events have demonstrated that even in countries with well-developed capacities related to food safety, unsafe foods that are produced abroad and imported for domestic consumption have the potential to result in large-scale outbreaks of foodborne disease. For example, nearly 4000 people became infected with *E.coli* (and nearly 800 developed haemolytic uremic syndrome) in Germany following the consumption of contaminated fenugreek sprouts, imported from Egypt in 2011. Illnesses related to the same imported product were concurrently reported in France.[6] In 2012, at least 11,000 cases of norovirus infection were reported in Germany following the consumption of frozen strawberries imported from China.[7] In 2008, 300,000 infants and children became ill in China, six of whom died, after consuming milk products contaminated with melamine. The contaminated products were directly exported or secondarily distributed to 47 countries around the world.[8] In 2013 and 2014, nearly 1,500 cases of hepatitis A infection were identified in 13 European countries and linked to the consumption of internationally distributed frozen berries.[9] More recently, in 2017 and 2018 the world bore witness to the largest outbreak of listeriosis on record which occurred in South Africa and resulted in more than 1000 cases

For peer review only - http://bmjopen.bmj.com/site/about/guidelines.xhtml

and 200 deaths. This protracted outbreak was eventually linked to domestically produced ready-to-eat meat products which were exported to 15 other countries in Africa.[10] Also in 2017 and 2018, an outbreak of salmonellosis in France affecting 37 infants was linked to contaminated infant formula that was exported worldwide to more than 80 countries.[11] These examples represent some of the largest international food safety events that have occurred in the recent past, either in terms of case-counts or number of countries affected, but smaller-scale events occur regularly. Furthermore, these events illustrate that even the most advanced food safety systems do not eliminate all foodborne hazards from reaching the public. The globalisation of our food supply means that unsafe food produced in one country can certainly result in cases of foodborne disease abroad.

Global food trade grew almost threefold from 2005 to 2015[12] and is projected to continue to rise.[13] Thus, there is a need for international coordination to facilitate rapid and efficient communication and collaboration between public health and food safety authorities (i.e. competent authorities) worldwide to prevent, detect and respond to international food safety events when internationally traded food is deemed unsafe. Until relatively recently, timely mechanisms to facilitate such global communication did not exist. In the early 2000s, WHO Member States recognised this gap and adopted resolutions at the World Health Assemblies in 2000[14] and 2002[15] calling for improved communication and coordination during international food safety events, including better tools to facilitate this. Since then, advancements in communication technology have facilitated the development or expansion of international networks and knowledge sharing platforms to exchange molecular subtyping information of foodborne pathogens, epidemiologic information about foodborne diseases, as well as information on food contamination and related traceability details. Throughout this protocol, the term 'communication tool' will be used to encompass networks, knowledge sharing platforms, technical programmes, or systems that facilitate communication related to food safety across national borders. These communication tools are complex for several reasons, including because they represent disparate systems that may or may not interface with each other, operate in different languages, are coordinated by different institutions in different countries and are at various

For peer review only - http://bmjopen.bmj.com/site/about/guidelines.xhtml

stages of development. Evidence from practice suggests that such tools are only effective within certain contexts and only cover certain geographic areas. [16, 17, 18, 19]. It is therefore necessary to unpack and explore the mechanisms of how and in what context such communication tools and their components are effective to facilitate international communication and coordination. Some examples of these communication tools include the European Rapid Alert System for Food and Feed (RASFF), the Association of Southeast Asian Nations (ASEAN) RASFF, and the International Food Safety Authorities Network (INFOSAN). The European RASFF system is an example of a regional tool that works in the European context, in part because member-countries adhere to the same legislation. The ASEAN RASFF system is an example of a tool that is less well established and member-countries in this Asian context do not adhere to the same legislation. INFOSAN is a global tool, coordinated by the World Health Organization (WHO) and the Food and Agriculture Organization of the United Nations (FAO), but as described by Savelli, Bradshaw, Ben Embarek, and Mateus[20], a relatively limited number of active members from a select group of countries contribute most information exchanged through the network. Supplementary file 1 provides a preliminary inventory of communication tools currently used or under development for exchanging information during international food safety events.

Unfortunately, a paucity of research has been conducted to investigate the attributes and effectiveness of the tools to facilitate cross-border communication during international food safety events. To date, most of the publications mentioning such tools focus on summarising a specific incident response, rather than explicitly examining the tools that were utilised. However, it is rather common for such reports of international food safety events to conclude by recommending that international efforts to strengthen rapid and efficient information exchange be improved through the further enhancement or utilisation of existing international networks and communication tools. These papers are typically written as outbreak reports rather than research studies.[8, 21-29] Such reports also commonly refer to context specific factors that facilitate or prevent rapid communication on various aspects of food safety investigations, such as poorly developed food safety systems, lack of national

For peer review only - http://bmjopen.bmj.com/site/about/guidelines.xhtml

coordination, or limited technical capacity. Available research provides limited guidance for decision-makers coordinating international programmes that facilitate information exchange on food safety, on how to adopt best practices to achieve their objectives. In addition, as explained by Savelli et al[20], the global food safety community would benefit from a thorough mapping of the interlinkages between such programmes and networks to better understand how they are being used, by whom and in what contexts. A realist synthesis is therefore proposed to begin to address this gap. The main question to guide this research is: how do different tools facilitate cross-border communication during international food safety events, why are they used, by whom, and for what purpose?

In this review, the proximal outcome of interest is the use of different tools to communicate internationally about issues related to food safety in an efficient manner. The distal outcomes of interest can be understood as the outcomes or consequences of using the tools. Some examples may include the identification of the source of an outbreak, facilitation of risk management actions in different countries, and prevention of foodborne disease. Although important, it is beyond the scope of this review to examine and measure the impact that using different tools has on the overall safety of the global food supply. However, several insights relating to the utility of different tools to prevent or mitigate the burden of foodborne disease will be garnered from this review to be further explored in future research. The terminology used in the review is outlined in supplementary file 2.

Research aim and objectives

The primary aim of this synthesis is to address the question: how do different tools facilitate cross-border communication during international food safety incidents, why are they used, by whom, and for what purpose? The overall objective is to refine a programme theory that explains the contexts (C) in which certain mechanisms (M) generate certain outcomes (O) by developing a series of C-M-O statements. This programme theory should prove useful to programme coordinators to promote and support the use of communication tools and improve their effectiveness. The specific objectives are as follows:

For peer review only - http://bmjopen.bmj.com/site/about/guidelines.xhtml

1) Document the different tools used to facilitate cross-border communication during international food safety incidents;
2) Examine the outcomes observed in relation to the use of different communication tools;
3) Identify and explain the mechanisms that influence the outcomes observed in relation to the use of different communication tools;
4) Identify the contextual factors that trigger mechanisms to influence the outcomes observed in relation to the use of different communication tools; and
5) Refine a realist programme theory that synthesises review findings and input from an expert reference committee to explain how different tools facilitate cross-border communication during international food safety events, why they are used, by whom, and for what purpose.

The realist approach

A realist approach has been chosen to conduct this review as it is well suited for the examination of complex programmes through its focus on outcomes in real-world settings and the contextual factors that influence them.[30] A realist perspective of social change underpins this approach whereby the actions of individuals and their understanding of the world serve to construct social phenomena and are influenced by cultural, institutional and social structures.[31, 32] This interpretative method is theoretically driven and allows evidence from a range of study designs to be synthesised. The use of theory facilitates a deeper understanding with respect to policy intentions and appreciates the complexity of programmes by including context in the analysis.[33] The overall intent of a realist review is the development and refinement of programme theories to understand how context influences mechanisms to generate outcomes. Mechanisms can be understood as the underlying context-dependent processes, behaviours, structures, values or levers that are able to generate outcomes. The context includes the social, cultural, institutional, historical and environmental factors that form the setting in which actions are taken to trigger mechanisms. The resulting outcomes of the

For peer review only - http://bmjopen.bmj.com/site/about/guidelines.xhtml

programme, system or intervention under examination are the products of certain mechanisms being triggered in certain contexts and may be intended or unintended.[30, 34, 35]

In this review, identifying mechanisms will help to explain how competent authorities utilise existing communication tools during international food safety events to exchange information across national borders. Taking the realist perspective, several C-M-O configurations may be articulated within the programme theory to explain this phenomenon. The C-M-O configurations will allow the research to be abstracted and applied to multiple contexts, bolstering external validity. The process of theory building and configuring the C-M-O will be iterative, enabling the confirmation, refutation or modification of the initial programme theory.[30, 33, 36]

Preliminary work to identify initial programme theory

To identify an initial programme theory, a range of sources have been utilised including the author's experiences as the current secretariat of the FAO/WHO International Food Safety Authorities Network (INFOSAN), a scoping review of published papers describing international food safety events and grey literature pertaining to various food safety communication tools currently in use and elicitation of input from an international expert reference committee consisting of 9 members including some coordinators of international communication tools currently in use (see acknowledgement section for details). This preliminary work has proposed an initial programme theory to suggest that when the context is such that a country: 1) is an importer or exported of food commodities; 2) has the technical infrastructure to detect food safety events (including foodborne disease outbreaks or food contamination); and 3) is governed in accordance with regional and/or global laws and regulations relating to food control and global health security, then certain mechanisms including trust, experience, support, awareness, understanding and a sense of community will facilitate the proximal outcome of using communication tools to relay information abroad and a potential range of distal outcomes, including: 1) intersectoral collaboration among different national stakeholders from agriculture, food and health authorities; 2) efficient exchange of information

For peer review only - http://bmjopen.bmj.com/site/about/guidelines.xhtml

between international stakeholders; 3) timely detection, notification, and response to food safety events (including the implementation of risk management measures); 4) reduction of food safety risks; 5) robust understanding of the international dimensions of a given food safety event; and 6) prevention of foodborne disease. It is proposed that variations in the context will influence whether the proposed mechanisms will trigger the outcomes. A schematic overview of this initial programme theory is provided in Figure 1.

Methods/design

This synthesis will adhere to the 2005 protocol provided by Pawson, Greenhalgh, Harvey, and Walshe for conducting realist reviews and reporting will be guided by the Realist and Meta-narrative Evidence Synthesis: Evolving Standards (RAMESES) from Wong, Greenhalgh, Westhorp, Buckingham, and Pawson.[33] The five steps for conducting a realist review according to Pawson et al.[30] will be followed: 1) clarify scope; 2) search for evidence; 3) appraise primary studies and extract data; 4) analyse and synthesise evidence; and 5) disseminate. While presented sequentially, these steps are iterative and will be revisited throughout the review process when new evidence emerges that can contribute to theory refinement. The grand level development theories that provide an overarching framework for this review include the third wave of modernisation theory developed in the 1990s [37, 38] and globalisation theory as articulated by Robinson.[39] Both theories provide a lens through which to understand that while the world is becoming ever more interconnected and interdependent, certain structures built to support development cannot be imposed in exactly the same way at the same time in different countries because the country-specific context will influence the outcomes. Modernisation theory also helps to explain the development of systems and tools within societies. This is particularly relevant in the context of ensuring food safety as there are international food safety standards and guidelines (including guidelines for communication during international food safety events) that must be adopted in national settings to improve food safety systems and facilitate food trade. Globalization theory helps to explain that with the introduction of international food safety standards and guidelines, national governments cannot operate in isolation if they wish to

For peer review only - http://bmjopen.bmj.com/site/about/guidelines.xhtml

engage in food trade. With this understanding and using the realist approach, a refined programme theory will be developed to explain context-mechanism-outcome (C-M-O) configurations related to the use of communication tools to facilitate information exchange during international food safety events. Two reviewers will undertake this work and the expert reference committee will provide feedback during the review. The review will be conducted over a 12-month period from January 2019 to December 2019 (see Figure 2 for an overview of the stages of this review).

Search strategy

To test the initial programme theory, a systematic search of the literature will aim to identify documents written in English, dating back to 1995 that illuminate how different tools facilitate cross-border communication during international food safety events, why they are used, by whom, and for what purpose. This search will be undertaken using the databases Web of Science, Embase, MEDLINE, PubMed and CINAHL. A comprehensive search algorithm has been developed with assistance from a librarian at Lancaster University, United Kingdom, by first selecting key search terms following the review of titles and abstracts from 10 known publications describing international food safety events or an international food safety communication tool, system or network. Combinations of the following key words in English (and their truncations where required) using Boolean operators and proximity operators (where possible) will be entered into the selected databases: (systems OR network OR tool OR communication OR notification OR "information exchange") AND (international OR multi-state OR multi-country OR imported OR exported) AND ((("food safety" OR "food contamination" OR "foodborne diseases") OR (gastroenteritis AND (incident OR emergency OR outbreak)) OR (food AND (incident Or emergency OR outbreak))). See supplementary file 3 for the specific database searches.

Bibliographic references from documents selected for inclusion will be reviewed using the snowballing method to identify other potentially relevant documents. Since grey literature can be a pertinent source of information for realist reviews, annual reports, evaluation summaries, or policy

For peer review only - http://bmjopen.bmj.com/site/about/guidelines.xhtml

documents published by international organisations or government agencies will also be searched for on respective websites.[30] The grey literature search will be purposeful and undertaken on the organizational websites related to those tools that have been already identified during the scoping review or through discussions with the expert reference committee, or that are later identified following the database searching. Members of the expert reference committee will also be asked to provide any grey literature pertaining to such tools they believe may be relevant. The search for evidence will be driven by the research objectives and will be iterative in practice to identify all relevant information sources to develop the programme theory. Searching will conclude when theoretical saturation is reached and sufficient evidence has been collected to confidently assert that the proposed theory is plausible.[33] The expert reference committee will contribute to this research by identifying additional articles and documents for consideration in the review and will provide feedback on the emerging programme theories as they are developed and refined. The search strategy will also be reviewed iteratively by this committee to ensure the scope of the search is appropriately designed to achieve the overall research aim and objectives. Throughout this process, references will be managed using Endnote X7 software.

Study selection criteria and procedures

To ensure that the development of programme theory considers a wide range of evidence, it is customary to utilise broad inclusion/exclusion criteria in a realist synthesis.[30] The inclusion criteria are studies of any design from peer-reviewed literature and other documents from grey literature that are written in English, published in 1995 or later, describe an international food safety event or a communication tool, and provide evidence that contributes to the synthesis and the emerging programme theory. The year 1995 was chosen because tools utilized prior to this are more likely to reference outdated technology (e.g. facsimile) that would not be relevant in today's internet-dependent world. The exclusion criteria are if a document does not describe an international food safety event or a communication tool with sufficient details to inform the programme theory or focuses on an outdated communication technology (e.g. facsimile). Two reviewers will independently

For peer review only - http://bmjopen.bmj.com/site/about/guidelines.xhtml

screen the title and abstract of the searched studies using the inclusion and exclusion criteria to maintain rigour in this review. If it is unclear from the title and abstract if a paper should be included (or if the paper does not have an abstract as with many documents from grey literature), the full text will be reviewed prior to exclusion. Differences will be discussed by the two reviewers and disagreements will be resolved through discussion with the expert reference committee until consensus is reached. This process will facilitate dialogue among reviewers and the expert reference committee in an effort to include all relevant data.

Data extraction and study appraisal

In realist synthesis, data extraction is more akin to note taking.[30] Each document included in the study will be reviewed using a bespoke data extraction form in Microsoft Excel to facilitate and organise note taking (supplementary file 4). The variables extracted will include: 1) title; 2) authors; 3) year of publication; 4) type of document/study 5) countries involved; 6) international/regional organisations involved; 7) specific foodborne hazard; 8) implicated food item; 9) name and details of communication tool used; 10) contextual factors that facilitated the use of the tool; 11) contextual factors that limited the use of the tool; 12) conclusions made by the authors with respect to the use of the tools; 13) recommendations made by the authors with respect to improving international communication during international food safety events; 14) any other contextual factors; 15) any other underlying mechanisms; and 16) Points of discussion to raise with expert reference committee. The use of this form is intended to focus on the extraction of information about contexts, mechanisms and outcomes on that which specifically contributes to the refinement of the initial programme theory.

As per the RAMESES guidelines, the quality appraisal will be made on the basis of how each study contributes to the development of C-M-O configurations. [33] In a realist synthesis, quality is determined by assessing two criteria: 1) relevance; and 2) rigour.[40] Relevance refers to the degree to which the information in the study fits within the scope of the review and rigor refers to methodological rigor and the degree to which conclusions reached in the study are appropriately

For peer review only - http://bmjopen.bmj.com/site/about/guidelines.xhtml

drawn based on the research design employed.[30] To aid in this assessment, the Mixed Methods Appraisal Tool[41] will be utilised, but will only be applied to the relevant aspects of each study under review and not necessary the whole study. This tool allows for assessment of multiple study designs concurrently, it has theoretical and content validity and it has also been tested for efficiency and reliability.[41, 42]. To assess relevance, each document will be scored as one of the following categories (adopted from Wozney et al[43] and Flynn et al[44]): 1) low/no contribution; 2) medium contribution; or 3) high contribution. Evidence will also be assessed as either objective (empirical) or subjective (anecdotal). The relevance and rigor of each of the included studies will be evaluated by two reviewers who will document a summary of their assessment in tabular format for consideration during analysis. Differences will be discussed by the two reviewers and disagreements will be resolved through discussion with the expert reference committee until consensus is reached. Documents will not be excluded based on the MMAT score, nor will documents from which evidence is anecdotal, but collecting this information will provide insight into the rigor of existing research in this field.

Data synthesis

With consideration for abductive and retroductive analysis,[45, 46] documents will be examined for evidence that support, refute or refine the initial programme theory. Synthesis will involve analysing data that were absent from the initial programme theory (abduction) and moving between theory and observable data (retroduction), enabling the formation of new ideas beyond the initial programme theory. Taking this approach will utilize both inductive and deductive analytic processes to understand the C-M-O configurations. A thematic approach will be applied to record patterns in context, mechanisms and outcomes within each document reviewed and then across documents. These patterns will be compared to the original programme theory to determine if they support, expand or refute its configuration. As articulated in the RAMESES guidelines, the intention here will be to interrogate the C-M-O configurations and not to provide quantifiable summary data from the studies reviewed.[21] If the reviewed data do not fully explain the initial theory or if new theories

For peer review only - http://bmjopen.bmj.com/site/about/guidelines.xhtml

emerge through this process, the literature search will be refocused in order to adequately synthesise a final programme theory with supporting thematic explanations.

Validity

Using an iterative approach to understand how different tools facilitate cross-border communication during international food safety events, why they are used, by whom, and for what purpose, will allow researchers to revisit the C-M-O configurations throughout the process as data from the literature is collected.[22] This practice and the intentional inclusion of context in the analysis will improve external validity and the potential generalisability of mechanisms identified in the review.[21] Further, the utilisation of an expert reference committee to elicit feedback, identify additional publications and review the programme theories as they are developed, serves to further bolster internal validity.

Ethics and Dissemination

Ethical approval is not required for this review as it does not involve primary research. However, it will be conducted according to the appropriate ethical standards of accuracy, utility, usefulness, accountability, feasibility and propriety.[32]

The RAMESES publication standards will be followed to report the findings of this review. Upon completion, the final manuscript will be shared with members of INFOSAN, which includes public health and food safety professionals from national government agencies in 188 countries. Further, it is the intent of the author to submit the review for publication in a leading peer-reviewed journal focusing on globalization and health. The review will also be submitted as a chapter in the first author's PhD thesis to be submitted to Lancaster University.

Patient and Public Involvement

For peer review only - http://bmjopen.bmj.com/site/about/guidelines.xhtml

For peer review only

Patients were not involved in the design of this study. As described earlier, the first author has involved an international group of experts to develop and refine the initial programme theory presented in this protocol.

Discussion

Significance

Increasingly, globalisation of our food supply necessitates international communication and coordination among food safety and public health professionals to prevent, detect, and respond to foodborne disease outbreaks and instances of food contamination that affect more than one country. Rigorous research is needed to understand how the various tools used to facilitate communication are working and in what contexts. The knowledge gained from this study will provide valuable lessons on how different tools facilitate cross-border communication during international food safety events, why they are used, by whom, and for what purpose.

Limitations

One limitation of this review is that it will only be conducted in English and therefore may introduce an element of language bias. In addition, the formulation of the context-mechanism-outcome programme theory relies heavily on published literature and therefore may be subject to publication bias. Finally, review findings will be context-specific and therefore must be considered within the context of this research.

Conclusion

Responding to international food safety events is complex, in part because of the globalised nature of our food supply and the involvement of numerous international stakeholders. In this paper, a protocol for conducting a realist synthesis on different tools to facilitate cross-border communication during international food safety events has been presented which has important but under-studied

For peer review only - http://bmjopen.bmj.com/site/about/guidelines.xhtml

implications on global efforts to mitigate the burden of foodborne illness resulting from internationally distributed food. The programme theory to be developed will be useful to policy makers and those coordinating the operation of communication tools currently in use, who may adapt components of the tools according to different contextual factors to promote, support and improve their use. By improving international coordination and communication during international food safety events, the global burden of foodborne disease can be mitigated.

Acknowledgements: The authors wish to acknowledge the members of expert review committee for their input during the development of the initial programme theory and for reviewing earlier drafts of this manuscript. Members include: Mr Jan Baele (RASFF Coordinator, European Commission, Belgium), Mr Juan Ortuzar (International Affairs Coordinator, Chilean Agency for Food Safety and Quality, Chile), Dr Caroline Merten (Scientific Officer, European Food Safety Authority, Italy), Ms Jenny Bishop (Team Manager, Food Compliance Services Group, Operations Branch, Ministry for Primary Industries, New Zealand), Dr Jorgen Schlundt (Director, Nanyang Technological University Food Technology Center, Singapore), Dr Peter Ben Embarek (Scientist, INFOSAN Secretariat, Department of Food Safety and Zoonoses, WHO, Switzerland), Mr Adam Bradshaw (Technical Officer, INFOSAN Secretariat, Department of Food Safety and Zoonoses, WHO, Switzerland), Mr Raul Garcia (Consultant, INFOSAN Secretariat, Department of Food Safety and Zoonoses, WHO, Switzerland) and Dr Peter Gerner-Smidt (Chief, Enteric Diseases Laboratory Branch, Centers for Disease Control and Prevention, USA).

For peer review only

Authors' contributions: CS conceived the original idea, designed the study, drafted the manuscript and approved the final document. CM drafted the manuscript and approved the final document. CS is a staff member of the World Health Organization. The author alone is responsible for the views expressed in this publication and they do not necessarily represent the views, decisions or policies of the World Health Organization.

Funding statement: This study is funded by the World Health Organization.

Competing interests: None

References

1. World Health Organization. (2015a). *Food safety*. Retrieved from: http://www.who.int/mediacentre/factsheets/fs399/en/

2. World Health Organization. (2015b). *Global burden of foodborne disease report*. Retrieved from: http://apps.who.int/iris/bitstream/10665/200046/1/WHO_FOS_15.02_eng.pdf?ua=1

3. World Health Organization. (2014). *Advancing food safety initiative: strategic plan for food safety including foodborne zoonoses 2013-2022*. Retrieved from: http://www.who.int/foodsafety/publications/strategic-plan/en/

4. World Bank. (2018). *The Safe Food Imperative : Accelerating Progress in Low- and Middle-Income Countries.* Retrieved from: https://openknowledge.worldbank.org/handle/10986/30568

5. World Health Organization. (2018a). *Food safety Data by WHO region*. Retrieved from: http://apps.who.int/gho/data/view.main.IHRREG11v?lang=en

6. Robert Koch Institute. (2011). *Technical Report EHEC/HUS O104:H4 Outbreak.*Germany, May/June 2011. Retrieved from: http://www.rki.de/EN/Home/EHEC_Report.pdf?__blob=publicationFile

7. Bernard, H., Faber, M., Wilking, H., Haller, S., Höhle, M., & Schielke, A. et al. (2014). Large multistate outbreak of norovirus gastroenteritis associated with frozen strawberries, Germany, 2012. *Eurosurveillance*, *19*(8), 20719. http://dx.doi.org/10.2807/1560-7917.es2014.19.8.20719

8. Gossner, C., Schlundt, J., Ben Embarek, P., Hird, S., Lo-Fo-Wong, D., & Beltran, J. et al. (2009). The Melamine Incident: Implications for International Food and Feed Safety. *Environmental Health Perspectives*, *117*(12), 1803-1808. http://dx.doi.org/10.1289/ehp.0900949

9. Severi, E., Verhoef, L., Thornton, L., Guzman-Herrador, B., Faber, M., & Sundqvist, L. et al. (2015). Large and prolonged food-borne multistate hepatitis A outbreak in Europe associated with consumption of frozen berries, 2013 to 2014. *Eurosurveillance*, *20*(29), 21192. http://dx.doi.org/10.2807/1560-7917.es2015.20.29.21192

10. World Health Organization. (2018b). *Listeriosis – South Africa*. Retrieved from: http://www.who.int/csr/don/28-march-2018-listeriosis-south-africa/en/

11. World Health Organization. (2018c). *INFOSAN Quarterly Summary*. Retrieved from: http://www.who.int/foodsafety/areas_work/infosan/INFOSAN-QS/en/

12. Food and Agriculture Organization of the United Nations. (2015). *The State of Agricultural Commodity Markets.* Retrieved from: http://www.fao.org/3/a-i5090e.pdf

13. International Food Policy Research Institute. (2018). 2018 Global Food Policy Report. Retrieved from: http://ebrary.ifpri.org/utils/getfile/collection/p15738coll2/id/132273/filename/132488.pdf

14. World Health Organization. (2000). *Resolution WHA53.15. Food Safety*. Retrieved from: http://apps.who.int/gb/archive/pdf_files/WHA53/ResWHA53/15.pdf

For peer review only - http://bmjopen.bmj.com/site/about/guidelines.xhtml

15. World Health Organization. (2002). *Resolution WHA55.16. Global public health response to natural occurrence, accidental release or deliberate use of biological and chemical agents or radionuclear material that affect health Food Safety*. Retrieved from: http://apps.who.int/gb/archive/pdf_files/WHA55/ewha5516.pdf

16. Food and Agriculture Organization of the United Nations/World Health Organization. (2013). *INFOSAN Activity Report 2011/2012*. Retrieved from: http://www.who.int/foodsafety/publications/activity-report-2012/en/

17. Food and Agriculture Organization of the United Nations/World Health Organization. (2014). *INFOSAN Activity Report 2013*. Retrieved from: http://www.who.int/foodsafety/publications/activity-report-2013/en/

18. Food and Agriculture Organization of the United Nations/World Health Organization. (2016). *INFOSAN Activity Report 2014/2015*. Retrieved from: http://apps.who.int/iris/bitstream/10665/246204/1/9789241510592-eng.pdf

19 Food and Agriculture Organization of the United Nations/World Health Organization. (2018). *INFOSAN Activity Report 2016/2017*. Retrieved from: https://www.who.int/foodsafety/publications/INFOSAN_Activity_Report_2016-17_EN.PDF?ua=1

20. Savelli, C., Bradshaw, A., Ben Embarek, P., Mateus, C. (2019). The FAO/WHO International Food Safety Authorities Network (INFOSAN) in Review, 2004-2018: Learning from the past and looking to the future. *Foodborne Pathogens and Disease.*

21. Guzman-Herrador, B., Nilsen, E., Cudjoe, K., Jensvoll, L., Kvamme, J., & Lindegård Aanstad, A. et al. (2013). A Shigella sonnei outbreak traced to imported basil – the importance of good typing tools and produce traceability systems, Norway, 2011. *Eurosurveillance*, 18(49), 20650. http://dx.doi.org/10.2807/1560-7917.es2013.18.49.20650

22. Khardori, N. (2012). A Multistate Outbreak of Hepatitis A Associated With Semidried Tomatoes in Australia, 2009. *Yearbook Of Medicine*, 2012, 101-102. http://dx.doi.org/10.1016/j.ymed.2012.08.004

23. Knoblauch, A., Bratschi, M., Zuske, M., Althaus, D., Stephan, R., Hächler, H., Baumgartner, A., Prager, R., Rabsch, W., Altpeter, E., Jost, M., Mäusezahl, M., Hatz, C. and Kiefer, S. (2015). Cross-border outbreak of Salmonella enterica ssp. enterica serovar Bovismorbificans: multiple approaches for an outbreak investigation in Germany and Switzerland. *Swiss Medical Weekly*.

24. Rebolledo, J., Garvey, P., Ryan, A., O'donnell, J., Cormican, M., & Jackson, S. et al. (2013). International outbreak investigation of Salmonella Heidelberg associated with in-flight catering. *Epidemiology And Infection*, 142(04), 833-842. http://dx.doi.org/10.1017/s0950268813001714

25. Einöder-Moreno, M., Lange, H., Grepp, M., Osborg, E., Vainio, K., & Vold, L. (2016). Non-heat-treated frozen raspberries the most likely vehicle of a norovirus outbreak in Oslo, Norway, November 2013. *Epidemiology And Infection*, 144(13), 2765-2772. http://dx.doi.org/10.1017/s0950268816000194

26. Inns, T., Ashton, P., Herrera-Leon, S., Lighthill, J., Foulkes, S., & Jombart, T. et al. (2016). Prospective use of whole genome sequencing (WGS) detected a multi-country outbreak of

For peer review only - http://bmjopen.bmj.com/site/about/guidelines.xhtml

Salmonella Enteritidis. *Epidemiology And Infection*, 145(02), 289-298. http://dx.doi.org/10.1017/s0950268816001941

27. Nygård, K., Lassen, J., Vold, L., Andersson, Y., Fisher, I., & Löfdahl, S. et al. (2008). Outbreak of Salmonella Thompson Infections Linked to Imported Rucola Lettuce. *Foodborne Pathogens And Disease*, 5(2), 165-173. http://dx.doi.org/10.1089/fpd.2007.0053

28. Pezzoli, L., Elson, R., Little, C., Yip, H., Fisher, I., & Yishai, R. et al. (2008). Packed with Salmonella —Investigation of an International Outbreak of Salmonella Senftenberg Infection Linked to Contamination of Prepacked Basil in 2007. *Foodborne Pathogens And Disease*, 5(5), 661-668. http://dx.doi.org/10.1089/fpd.2008.0103

29. Acciari, V., Iannetti, L., Gattuso, A., Sonnessa, M., Scavia, G., Montagna, C., Addante, N., Torresi, M., Zocchi, L., Scattolini, S., Centorame, P., Marfoglia, C., Prencipe, V. And Gianfranceschi, M. (2015). Tracing sources of Listeria contamination in traditional Italian cheese associated with a US outbreak: investigations in Italy. *Epidemiology and Infection*, 144(13), pp.2719-2727.

30. Pawson, R., Greenhalgh, T., Harvey, G., & Walshe, K. (2005). Realist review - a new method of systematic review designed for complex policy interventions. *Journal Of Health Services Research & Policy*, *10*(1_suppl), 21-34. http://dx.doi.org/10.1258/1355819054308530

31. Gunderson, L. and Holling, C. (2002). *Panarchy*. (1st ed). Washington, DC: Island Press.

32. Mertens, D. (2009). *Transformative research and evaluation*. 1st ed. New York: Guilford Press.

33. Wong, G., Greenhalgh, T., Westhorp, G., Buckingham, J., & Pawson, R. (2013). RAMESES publication standards: realist syntheses. *BMC Medicine*, *11*(1). http://dx.doi.org/10.1186/1741-7015-11-21

34. Thompson, S., Clark, A., Molzahn, A., Klarenbach, S., & Tonelli, M. (2016). Increasing the uptake of exercise programs in the dialysis unit: a protocol for a realist synthesis. *Systematic Reviews*, *5*(1). http://dx.doi.org/10.1186/s13643-016-0224-6

35. Pitzul, K., Lane, N., Voruganti, T., Khan, A., Innis, J., Wodchis, W., & Baker, G. (2015). Role of context in care transition interventions for medically complex older adults: a realist synthesis protocol. *BMJ Open*, *5*(11), e008686. http://dx.doi.org/10.1136/bmjopen-2015-008686

36. Durham, J. & Blondell, S. (2014). Research protocol: a realist synthesis of cross-border patient mobility from low-income and middle-income countries. *BMJ Open*, *4*(11), e006514. http://dx.doi.org/10.1136/bmjopen-2014-006514

37. Giddens, A. (1990). *The consequences of modernity* (1st ed.). Stanford, Calif.: Stanford University Press.

38. Giddens, A. (1991). *Modernity and self-identity*. (1st ed). Stanford, Calif.: Stanford University Press.

39. Robinson, W. (2011). Globalization and the sociology of Immanuel Wallerstein: A critical appraisal. *International Sociology*, *26*(6), 723-745. http://dx.doi.org/10.1177/0268580910393372

For peer review only - http://bmjopen.bmj.com/site/about/guidelines.xhtml

40. Pawson, R. (2006). *Evidence based policy: a realist perspective*. London: Sage.

41. Hong QN, Pluye P, Fàbregues S, Bartlett G, Boardman F, Cargo M, Dagenais P, Gagnon M-P, Griffiths F, Nicolau B, O'Cathain A, Rousseau M-C, Vedel I. Mixed Methods Appraisal Tool (MMAT), version 2018. Registration of Copyright (#1148552), Canadian Intellectual Property Office, Industry Canada.

42. Pluye, P., Gagnon, M., Griffiths, F., & Johnson-Lafleur, J. (2009). A scoring system for appraising mixed methods research, and concomitantly appraising qualitative, quantitative and mixed methods primary studies in Mixed Studies Reviews. *International Journal Of Nursing Studies*, *46*(4), 529-546. http://dx.doi.org/10.1016/j.ijnurstu.2009.01.009

43. Wozney L, Huguet A, Newton A, et al. How do eHealth programs for adolescents with depression work? A realist review of persuasive system design components in internet-based psychological therapies. J Med Internet Res. 2017;19(8):e266. doi: 10.2196/jmir.7573

44. Flynn R, Newton A, Rotter T, Hartfield D, Walton S, Fiander M et al. The sustainability of Lean in pediatric healthcare: a realist review. Systematic Reviews. 2018;7(1).

45. Meyer BS, Lunnay B. The application of abductive and retroductive inference for the design and analysis of theory-driven sociological research. Sociol Res Online. 2013;18(1):1–11. doi: 10.5153/sro.2819.

46. Greenhalgh T, Pawson R, Wong G, Westhorp G, Greenhalgh J, Manzano A, Jagosh J. Retroduction in Realist Evaluation. 2017.

For peer review only - http://bmjopen.bmj.com/site/about/guidelines.xhtml

Figure legends/captions

Figure 1

Legend: N/A

Caption: To identify an initial programme theory, a range of sources have been utilised including the author's experiences as the current secretariat of the FAO/WHO International Food Safety Authorities Network (INFOSAN), a scoping review of published papers describing international food safety events and grey literature pertaining to various food safety communication tools currently in use and elicitation of input from an international expert reference committee including some coordinators of international communication tools currently in use.

Figure 2

Legend: N/A

Caption: The review will be conducted over a 12-month period from January 2019 to December 2019.

For peer review only

For peer review only - http://bmjopen.bmj.com/site/about/guidelines.xhtml

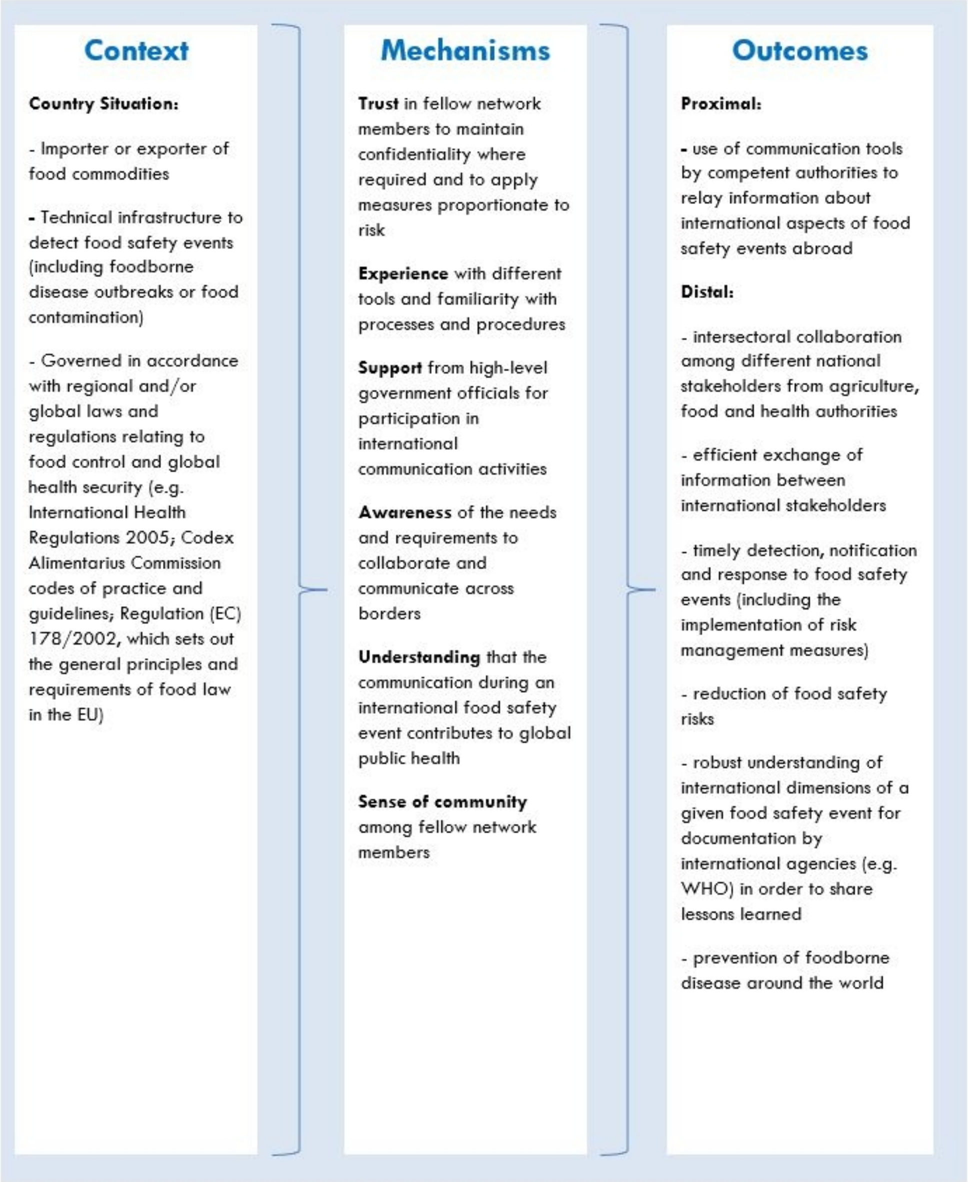

To identify an initial programme theory, a range of sources have been utilised including the author's experiences as the current secretariat of the FAO/WHO International Food Safety Authorities Network (INFOSAN), a scoping review of published papers describing international food safety events and grey literature pertaining to various food safety communication tools currently in use and elicitation of input from an international expert reference committee including some coordinators of international communication tools currently in use.

186x226mm (96 x 96 DPI)

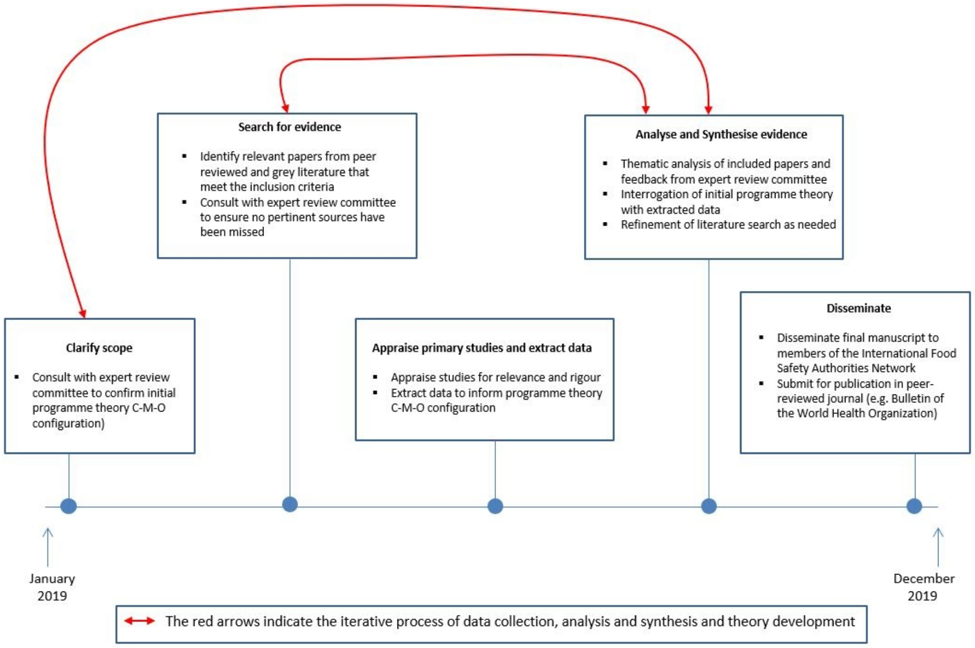

The review will be conducted over a 12-month period from January 2019 to December 2019.

287x214mm (96 x 96 DPI)

Supplementary File 1 – Preliminary inventory of communication tools currently used to exchange information during international food safety events*

Name of tool	Description	Type of Users	Primary Mode of Communication	Primary type of information exchanged	Coordinating Responsibly
International Food Safety Authorities Network<br><br>(INFOSAN)	Rapid sharing of information during food safety emergencies to stop the spread of contaminated food from one country to another; facilitates the sharing of experiences and tested solutions in and between countries in order to optimise future interventions to protect the health of consumers; launch in 2004	Officially designated government officials from multiple sectors involved in food safety (e.g. health, agriculture, veterinary services, trade, etc.); Members from 186 countries + European Commission (European Centre for Disease Control (ECDC), European Food Safety Authority (EFSA), Rapid Alert System for Food and Feed (RASFF) Commission Contact Point)	Web-based communication platform<br><br>(INFOSAN Community Website, members only)	Food contamination information along with export details; foodborne outbreak information including related epidemiologic, laboratory and traceability information	Secretariat at WHO; (Jointly managed by FAO and WHO)
For more information, see: http://www.who.int/foodsafety/areas_work/infosan/en/					
Rapid Alert System for Food and Feed<br><br>(RASFF)	Provides food and feed control authorities with an effective tool to exchange information about measures taken responding to serious risks detected in relation to food or feed; launched in 1979	Members from 28 national food safety authorities in the EU + EFSA, ESA, Norway, Liechtenstein, Iceland and Switzerland & WHO	Web-based communication platforms (RASFF Window, members only)	Food contamination information along with export details; foodborne outbreak information including related epidemiologic, laboratory and traceability information	Directorate General for Health and Food Safety<br><br>(DG SANTE, European Commission)
For more information, see: https://ec.europa.eu/food/safety/rasff_en					
Early Warning and Response system of the European Union<br><br>(EWRS)	Confidential computer system allowing Member States to send alerts about events with a potential impact on the EU, share information, and coordinate their response	Members from 28 Public Health Authorities of the EU Member States, which have been designated officially by the Government of their Country as members of the EWRS, and the competent service of the European Commission & WHO	Web-based communication platform (members only)	Foodborne disease surveillance information	ECDC<br><br>(European Commission)
For more information, see: http://www.eurosurveillance.org/ViewArticle.aspx?ArticleId=666					
Epidemic Intelligence Information System for Foodborne and Waterborne diseases and Zoonoses<br><br>(EPIS-FWD)	Facilitates the early detection and assessment of multi-country/multinational molecular typing clusters and outbreaks of Foodborne and Waterborne diseases and Zoonoses	The platform connects epidemiologists and microbiologists from 52 countries: 28 EU Member States, three countries of the European Economic Area (EEA) - Iceland, Norway and Liechtenstein - and 21 other non-EU countries & WHO	Web-based communication platform (members only)	Molecular typing clusters and foodborne, waterborne and Zoonotic outbreak details;	ECDC (European Commission)
For more information, see: http://ecdc.europa.eu/en/aboutus/what-we-do/epidemic-intelligence/Pages/EpidemicIntelligence_Tools.aspx					
Hepatitis A Lab-Network	Global network of hepatitis A reference laboratories	Scientists working in hepatitis A reference laboratories	Email; Web-based electronic database (members only)	Molecular and epidemiological data on hepatitis A virus	National Institute for Public Health and the Environment

For peer review only

(HAVNet)					(RIVM, The Netherlands)
For more information, see: http://www.eurosurveillance.org/ViewArticle.aspx?ArticleId=21356					
PulseNet International	Network of national and regional laboratory networks dedicated to tracking foodborne infections world-wide; Each laboratory utilises standardised genotyping methods, sharing information in real-time	Scientists working in public health laboratories and academic and medical institutions, including 10 countries in the Eastern Mediterranean, 16 countries in the Americas, 11 countries in the Western Pacific, 11 countries in Africa, 31 countries in Europe	PulseNet International forum hosted by PulseNet Canada (Web-based, members only, communication platform)	Genotyping information from foodborne pathogens (some genomic data)	US Centers for Disease Control (CDC)
For more information, see: http://www.pulsenetinternational.org/					
ASEAN Rapid Alert System for Food and Feed	Regional network modelled after the European RASFF system involving 10 ASEAN Member States.	Competent Authorities in Food Safety and Public Health of all ASEAN Member States (10 countries)	web-based application	Information on direct or indirect risks to human deriving from food or feed being traded in ASEAN and measures taken to prevent them entering the food chain	National Bureau of Agricultural Commodity and Food Standards (ACFS),<br><br>Ministry of Agriculture and Cooperatives, Thailand.
For more information, see: http://arasff.net/index.php					
Bi-lateral agreements	Many national authorities have established bi-lateral agreements with foreign agencies for the purpose of exchanging information during international food safety events.	National authorities.	Email or telephone.	Could be any type of information required to respond to an international food safety event.	National authorities.

*This list has been informed by the author's experience as the WHO secretariat of INFOSAN, the results from a scoping search, and input from the Expert Reference Committee

Supplementary file 2 - Definitions of key terms

Communication tools: Networks, knowledge sharing platforms, technical programmes, or systems that facilitate communication related to food safety across national borders.

Context: The context includes the social, cultural, institutional, historical and environmental factors that form the setting in which actions are taken to trigger mechanisms.

Context–mechanism–outcome configuration: A relationship between the elements of realist synthesis; how mechanisms are triggered under specific contexts to result in outcomes.

International Food Safety Events: An international food safety event results when unsafe food produced in one country, is exported to at least one other country (and may or may not result in an outbreak of foodborne illness).

Mechanism: Mechanisms can be understood as the underlying context-dependent processes, behaviours, structures, values or levers that are able to generate outcomes.

Outcome: The impact or behaviours resulting from the interaction between mechanisms and contexts.

Programme theory: A set of theoretical explanations or assumptions about how a particular programme, process or intervention is expected to work or cause the intended or observed outcomes.

For peer review only

Supplemental File 3 – Detailed Search Strategies for Web of Science, Embase, MEDLINE, PubMed and CINAHL

Web of Science

Search History:

Set	Results	Save History / Create Alert · Open Saved History
# 7	1,027	#6 AND #5 AND #4<br>*Indexes=SCI-EXPANDED, SSCI, A&HCI, CPCI-S, CPCI-SSH, ESCI Timespan=1995-2019*
# 6	9,703,534	TS=(systems OR network OR tool OR communication OR notification OR “information exchange”)<br>*Indexes=SCI-EXPANDED, SSCI, A&HCI, CPCI-S, CPCI-SSH, ESCI Timespan=1995-2019*
# 5	806,837	TS=(international OR multi-state OR multi-country OR imported OR exported)<br>*Indexes=SCI-EXPANDED, SSCI, A&HCI, CPCI-S, CPCI-SSH, ESCI Timespan=1995-2019*
# 4	32,199	#3 OR #2 OR #1<br>*Indexes=SCI-EXPANDED, SSCI, A&HCI, CPCI-S, CPCI-SSH, ESCI Timespan=1995-2019*
# 3	26,514	TS=("food safety" OR "food contamination" OR "foodborne diseases")<br>*Indexes=SCI-EXPANDED, SSCI, A&HCI, CPCI-S, CPCI-SSH, ESCI Timespan=1995-2019*
# 2	1,793	TS=(gastroenteritis NEAR/5 (incident* OR emergenc* OR outbreak*))<br>*Indexes=SCI-EXPANDED, SSCI, A&HCI, CPCI-S, CPCI-SSH, ESCI Timespan=1995-2019*
# 1	5,547	TS=(food* NEAR/5 (incident* Or emergenc* OR outbreak*))<br>*Indexes=SCI-EXPANDED, SSCI, A&HCI, CPCI-S, CPCI-SSH, ESCI Timespan=1995-2019*

Embase and MEDLINE

# ▲	Searches	Results
1	(food* adj5 incident*).af.	983
2	(food* adj5 emergenc*).af.	5617
3	(food* adj5 outbreak*).af.	8825
4	(gastroenteritis adj5 incident*).af.	54
5	(gastroenteritis adj5 emergenc*).af.	1206
6	(gastroenteritis adj5 outbreak*).af.	4424
7	("food safety" or "food contamination" or "foodborne diseases").af.	156116
8	(international or multi-state or multi-country or imported or exported).af.	3541525
9	(systems or network or tool or communication or notification or information-exchange).af.	4810679
10	1 or 2 or 3	15272
11	4 or 5 or 6	5640
12	7 or 10 or 11	170450
13	8 and 9 and 12	2835
14	limit 13 to yr="1995 -Current"	2779

PubMed

Search	Add to builder	Query	Items found	Time
#7	Add	Search ((((systems OR network OR tool OR communication OR notification OR "information exchange"))) AND ((international OR multi-state OR multi-country OR imported OR exported))) AND (((((("food safety" OR "food contamination" OR "foodborne diseases"))) OR ((gastroenteritis AND (incident* OR emergenc* OR outbreak*)))) OR ((food* AND (incident* Or emergenc* OR outbreak*))))	1062	10:27:02
#6	Add	Search (systems OR network OR tool OR communication OR notification OR "information exchange")	2269385	10:26:36
#5	Add	Search (international OR multi-state OR multi-country OR imported OR exported)	528235	10:26:03
#4	Add	Search (((("food safety" OR "food contamination" OR "foodborne diseases"))) OR ((gastroenteritis AND (incident* OR emergenc* OR outbreak*)))) OR ((food* AND (incident* Or emergenc* OR outbreak*)))	105011	10:25:24
#3	Add	Search ("food safety" OR "food contamination" OR "foodborne diseases")	74378	10:24:58
#2	Add	Search (gastroenteritis AND (incident* OR emergenc* OR outbreak*))	13879	10:24:38
#1	Add	Search (food* AND (incident* Or emergenc* OR outbreak*))	26356	10:24:21

CINAHL (with proximity operators; syntax: n5)

Search ID#	Search Terms	Search Options	Actions
S7	S4 AND S5 AND S6	Search modes - Find all my search terms	View Results (79) View Details Edit
S6	systems OR network OR tool OR communication OR notification OR "information exchange"	Search modes - Find all my search terms	View Results (836,810) View Details Edit
S5	international OR multi-state OR multi-country OR imported OR exported	Search modes - Find all my search terms	View Results (125,224) View Details Edit
S4	S1 OR S2 OR S3	Search modes - Find all my search terms	View Results (6,148) View Details Edit
S3	"food safety" OR "food contamination" OR "foodborne diseases"	Search modes - Find all my search terms	View Results (5,155) View Details Edit
S2	gastroenteritis N5 (incident* OR emergenc* OR outbreak*)	Search modes - Find all my search terms	View Results (417) View Details Edit
S1	food* N5 (incident* Or emergenc* OR outbreak*)	Search modes - Find all my search terms	View Results (889) View Details Edit

Supplemental File 4 - Data Extraction Form

EndNote Reference Number
**Title**
**Authors**
**Year of publication**
**Type of document/study**
**Countries involved (specify where food was produced, where food was distributed, and where illnesses occurred)**
**International/regional organizations involved (specify who took a coordinating role)**
**Specific foodborne hazard**
**Implicated food item (specify how food was implicated, e.g. epidemiologic evidence, laboratory evidence, traceability evidence)**
**Name and details of communication tool(s) used (including who used the tool and what kind of information was exchanged)**
**Factors that facilitated the use of the tools (context or mechanisms)**
**Factors that limited the use of the tool (context or mechanisms)**
**Conclusions made by the authors with respect to the use of the tools (related to context, mechanisms or outcomes)**
**Recommendations made by the authors with respect to improving international communication during international food safety events**
**Any other contextual factors**

For peer review only

Any other underlying mechanisms
**Points of discussion to raise with Expert Reference Committee**

For peer review only
